# An ENU mutagenesis screen identifies novel and known genes involved in epigenetic processes in the mouse

**DOI:** 10.1186/gb-2013-14-9-r96

**Published:** 2013-09-11

**Authors:** Lucia Daxinger, Sarah K Harten, Harald Oey, Trevor Epp, Luke Isbel, Edward Huang, Nadia Whitelaw, Anwyn Apedaile, Anabel Sorolla, Joan Yong, Vandhana Bharti, Joanne Sutton, Alyson Ashe, Zhenyi Pang, Nathan Wallace, Daniel J Gerhardt, Marnie E Blewitt, Jeffrey A Jeddeloh, Emma Whitelaw

**Affiliations:** 1 Epigenetics Laboratory, QIMR Berghofer Medical Research Institute, Herston, Qld 4006, Australia; 2 La Trobe Institute for Molecular Science, Department of Genetics, La Trobe University, Bundoora 3086, Vic, Australia; 3 Development and Research, Roche NimbleGen, 500 South Rosa Road, Madison, WI 53705, USA; 4 Molecular Medicine Division, The Walter and Eliza Hall Institute of Medical Research, University of Melbourne, Melbourne 3050, Vic, Australia; 5 Department of Medical Biology and Dept of Genetics, University of Melbourne, Melbourne 3050, Vic, Australia; 6 Present address: Institute of Molecular Genetics of ASCR, Videnska 1083, Prague 4, Czech Republic; 7 Present address: Gurdon Institute, University of Cambridge, Cambridge CB2 IQN, UK

## Abstract

**Background:**

We have used a sensitized ENU mutagenesis screen to produce mouse lines that carry mutations in genes required for epigenetic regulation. We call these lines *Modifiers of murine metastable epialleles* (*Mommes*).

**Results:**

We report a basic molecular and phenotypic characterization for twenty of the *Momme* mouse lines, and in each case we also identify the causative mutation. Three of the lines carry a mutation in a novel epigenetic modifier, *Rearranged L-myc fusion* (*Rlf*), and one gene, *Rap-interacting factor 1* (*Rif1*), has not previously been reported to be involved in transcriptional regulation in mammals. Many of the other lines are novel alleles of known epigenetic regulators. For two genes, *Rlf* and *Widely-interspaced zinc finger* (*Wiz*), we describe the first mouse mutants. All of the *Momme* mutants show some degree of homozygous embryonic lethality, emphasizing the importance of epigenetic processes. The penetrance of lethality is incomplete in a number of cases. Similarly, abnormalities in phenotype seen in the heterozygous individuals of some lines occur with incomplete penetrance.

**Conclusions:**

Recent advances in sequencing enhance the power of sensitized mutagenesis screens to identify the function of previously uncharacterized factors and to discover additional functions for previously characterized proteins. The observation of incomplete penetrance of phenotypes in these inbred mutant mice, at various stages of development, is of interest. Overall, the *Momme* collection of mouse mutants provides a valuable resource for researchers across many disciplines.

## Background

Mutagenesis screens for modifiers of position effect variegation in *Drosophila* have played a defining role in the development of the field of epigenetics [1]. The screens used a fly strain that showed variegated expression of the *white* (*w*) locus, resulting in red and white patches in the eye, as a result of the stochastic establishment of epigenetic state. The genes identified by these screens turn out to have pivotal roles in gene silencing [2-4]. We have designed a similar screen in the mouse, using a green fluorescent protein (GFP) transgene that shows variegated expression in red blood cells. Offspring of N-ethyl-N-nitrosourea (ENU)-treated males are screened for changes in the percentage of erythrocytes expressing GFP; this is a screen for dominant effects. Each mutant line is named a *Modifier of murine metastable epiallele Dominant*, *MommeD*[5]. The underlying mutations have been identified and published for nine lines and the mutations occur in DNA methyltransferases (*Dnmt1* and *Dnmt3b*), chromatin remodelers (*Smarca5* and *Baz1b*), a histone deacetylase (*Hdac1*), a transcriptional co-repressor (*Trim28*), a eukaryotic translation initiation factor (*eIF3h*) [6-10] and a previously unknown gene, *Smchd1*, now shown to be required for X-inactivation in the mouse [11,12]. Recently, *Smchd1* has been shown to act as a tumor suppressor [13] and mutations in SMCHD1 have been shown to be tightly associated with the human disease facioscapulohumeral dystrophy type 2 (FSHD2) [14].

## Results and discussion

### Identification of *Momme* mutants

We have now screened a total of approximately 5,000 G1 offspring, recovered 42 *MommeD* lines and identified the underlying mutations in 29 cases. These lines carry mutations in 18 unique genes (Table 1). A number of the genes have been hit more than once. Based on their effect on GFP expression, 14 lines were classified as suppressors of variegation, that is, the mutation increased the percentage of erythrocytes expressing GFP, and 15 lines were classified as enhancers of variegation, that is, the mutation decreased the percentage of erythrocytes expressing GFP (Table 1). Here we report, for the first time, the underlying mutations in 20 *MommeD* lines, which carry mutations in 10 unique genes.

**Table 1:** ***MommeD *** **mutants, causative mutations and disease association**

**Name**	**Effect on variegation**	**Gene**	**Nature of mutation**	**Homozygous phenotype**	**References**	**Human homolog**	**Disease association**
*MommeD1*	Suppressor	*Smchd1*	C->T exon 23; introduces Stop	Null	[5,11]	SMCHD1	FSHD2 [14]
*MommeD2*	Suppressor	*Dnmt1*	C->A exon 25; T812K	Null	[6]	DNMT1	Schizophrenia, breast and prostate cancer [15-19]
*MommeD4*	Enhancer	*Smarca5*	T->A exon 12; W520R	Hypomorphic?	[5,6]	SMARCA5	Acute myeloid leukemia [20]
*MommeD5*	Enhancer	*Hdac1*	7 bp deletion in exon 13; Frameshift	Null	[5,7]	HDAC1	Schizophrenia, neural development [2]
*MommeD8*	Enhancer	*Rlf*	G->T exon 8; C1558F	Hypomorphic	This study	RLF	
*MommeD9*	Enhancer	*Trim28*	T->C splice donor site of intron 13	Null	[8]	TRIM28	
*MommeD10*	Enhancer	*Baz1b*	T->G exon 7; L733R	Hypomorphic	[7]	BAZ1B	Williams-Beuren syndrome [21]
*MommeD12*	Enhancer	*eIF3h*	T->A - 10 bp before splice acceptor site of intron 4	Null	[9]	eIF3H	
*MommeD13*	Suppressor	*Setdb1*	A->G exon 20; results in splicing defect	Null	This study	SETDB1	Melanoma [22]
*MommeD14*	Suppressor	*Dnmt3b*	T->C splice acceptor site of intron 12	Hypomorphic	[10]	DNMT3B	ICF syndrome [23]
*MommeD16*	Enhancer	*Baz1b*	T->C exon 2; L75P	Hypomorphic?	This study	BAZ1B	Williams-Beuren syndrome [21]
*MommeD17*	Suppressor	*Setdb1*	T->C exon 21; V1248A	Hypomorphic	This study	SETDB1	Melanoma [22]
*MommeD18*	Suppressor	*Rif1*	C->T exon 29; Q1669 Stop	Null	This study	RIF1	Breast cancer [24]
*MommeD19*	Suppressor	*Smarcc1*	T->G splice acceptor site of intron 10	Null	This study	SMARCC1	Colorectal cancer [25,26]
*MommeD23*	Suppressor	*Smchd1*	A->T exon 12; R498 Stop	Null?	This study	SMCHD1	FSHD2 [14]
*MommeD27*	Suppressor	*Pbrm1*	A->G exon 17; Y733C	Hypomorphic?	This study	PBRM1	Renal cancer [27]
*MommeD28*	Enhancer	*Rlf*	A->G splice acceptor site of intron 4	Null	This study	RLF	
*MommeD30*	Enhancer	*Wiz*	1 bp deletion in exon 5; Frameshift at amino acid 553	Null	This study	WIZ	
*MommeD31*	Enhancer	*Trim 28*	T->A exon 3; C178S	Null?	This study	TRIM28	
*MommeD32*	Suppressor	*Dnmt1*	T->C exon 29; L1045P	Null?	This study	DNMT1	Schizophrenia, breast and prostate cancer [15-19]
*MommeD33*	Suppressor	*Suv39h1*	A->G splice donor site of intron 1	Null	This study	SUV39H1	Lupus, retinoblastoma [2]
*MommeD34*	Enhancer	*Rlf*	C->A exon 7; C355 Stop	Null	This study	RLF	
*MommeD35*	Enhancer	*Smarca5*	A-> G exon 9; N341S	Hypomorphic?	This study	SMARCA5	Acute myeloid leukemia [20]
*MommeD36*	Suppressor	*Smchd1*	C->T exon 42; Q1732 Stop	Null?	This study	SMCHD1	FSHD2 [14]
*MommeD37*	Enhancer	*Smarca5*	T->C exon 13; L565P	Null?	This study	SMARCA5	Acute myeloid leukemia [20]
*MommeD38*	Enhancer	*eIF3h*	G->A exon 7; R291 Stop	Null	[9]	eIF3H	
*MommeD39*	Suppressor	*Smarca4*	G->A splice donor site of intron 20	Null	This study	SMARCA4	Coffin-Siris syndrome [28]
*MommeD40*	Suppressor	*Uhrf1*	T->A exon17; Y778 Stop	Null	This study	UHRF1	
*MommeD42*	Enhancer	*Brd1*	T->A exon 11; C411 Stop	Null	This study	BRD1	Schizophrenia, bipolar affective disorder [29]

In some cases the mutation can be defined as hypomorphic or null and in other cases this designation is less certain and we have added a question mark.

**Figure 1:**
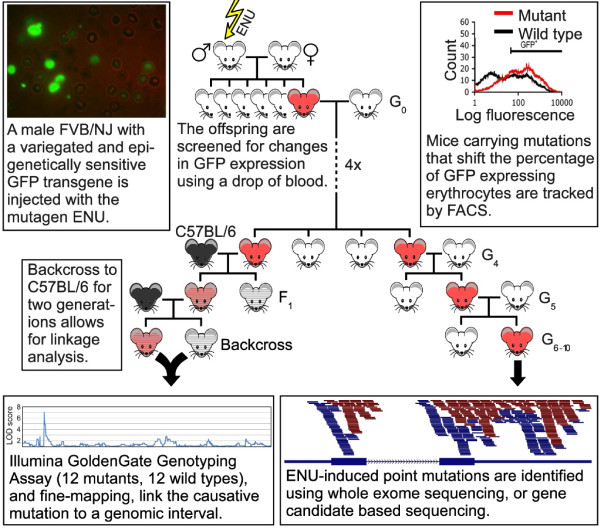
**An ENU mutagenesis/whole exome deep sequencing pipeline enables rapid identification of causative mutations in mice with defects in epigenetic gene silencing.** A schematic overview of the ENU mutagenesis gene discovery pipeline is presented. The major components of the screen are described in the figure. Briefly, male FVB/NJ mice carrying an epigenetically sensitive GFP transgene (*Line3*) were treated with ENU and mated with female *Line3* mice. Offspring were screened for a shift in the percentage of GFP expressing erythrocytes using flow cytometry. Putative mutants were mated with *Line3* mice for four generations to test for heritability and reproducibility of the GFP expression, and to reduce the number of non-causative ENU mutations within the genomes. Linkage analysis was carried out on the offspring from two generations of backcrosses between putative mutants and C57BL/6J mice, which also carry the GFP transgene (*Line3C*). Causative mutations were identified by whole exome deep sequencing, or gene candidate sequencing on individuals that had been maintained on the *Line3* background for at least seven generations. FACS, fluorescence-activated cell sorting.

The experimental pipeline for the screen is shown in Figure 1. In brief, ENU mutagenesis was carried out as described previously [5] and mapping was carried out following a G2 backcross to *Line3C* (a C57BL/6J strain carrying the same GFP transgene array at the same location) using traditional microsatellite and SNP genotyping or an Illumina GoldenGate SNP genotyping assay. Mapping intervals for the latter group are shown as Manhattan plots in Additional file 1.

To identify the underlying mutations, several different strategies have been used. In ten cases, the mutation was identified following whole exome deep sequencing of DNA from one mutant mouse from the *MommeD* inbred colony. For these, variants were called within the intervals linked to the causative mutation. For some lines, variant calling was extended to exome-wide, to assess for passenger mutations but only a small number was found (<10). These did not reflect the typical ENU sequence bias (T/A) [30] and probably represent normal background mutations.

In two instances, we have used a custom capture array to specifically enrich for DNA from a 4.2 Mbp interval previously identified by linkage analysis [7] followed by deep sequencing to identify the underlying mutations. In the remaining eight cases, Sanger sequencing of the exons (including splice sites) of candidate genes in the linked intervals was carried out. Details on identification of the mutations are provided in Materials and methods. In all cases, putative mutations were verified by PCR and Sanger sequencing in larger cohorts (at least 100 mice per mutant line).

In the two cases in which a custom capture array was used, we had an opportunity to obtain an estimate of the ENU mutation rate. In one case four mutations were found in the 4.2 Mbp captured interval and in the other case six mutations were found. Based on these results we estimated the ENU mutation rate at approximately 1 per Mbp. This is consistent with reports by others [31].

The heritability of the mutations was tested over at least six generations. Flow cytometric expression profiles and the percentage of erythrocytes expressing the transgene for each *MommeD* (presented here for the first time) are shown in Additional file 2.

### *MommeD30* mice are haploinsufficient for Widely interspaced zinc finger (Wiz)

**Figure 2:**
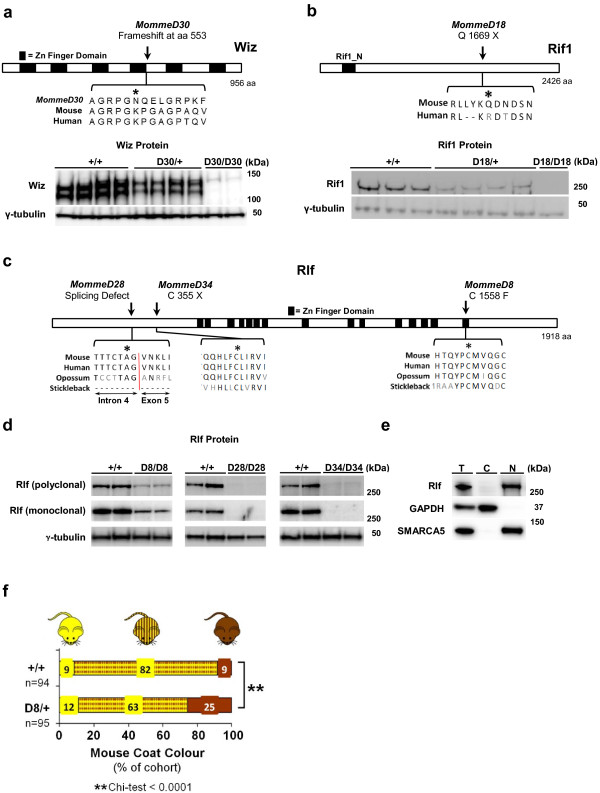
**Causative mutations in *MommeD30*, *MommeD18*, *MommeD8*, *MommeD28 *and *MommeD34*. (a)***MommeD30* carries a 1 bp deletion in *Wiz* that leads to a frame-shift at amino acid 553. Western blot analysis of embryo heads at 12.5 days post-coitum (dpc) shows reduced levels of Wiz protein in *Wiz*^*MommeD30*^ heterozygotes and no Wiz protein in homozygotes. Wiz protein was detected at approximately 120 and approximately 130 kDa. Each track represents a different animal. Tubulin was used as a loading control. **(b)***MommeD18* harbors a point mutation in *Rif1* that introduces a premature stop codon at amino acid 1,669. Western analysis of testes tissue shows that *Rif*^*MommeD18*^ heterozygotes have a reduced dosage of Rif1. No Rif1 protein was detected in a homozygote. Each track represents a different animal. Rif1 protein was detected at approximately 260 kDa and Tubulin was used as a loading control. **(c)** ENU point mutations in *MommeD8*, *MommeD28* and *MommeD34* occur in conserved regions of the Rlf protein. Asterisks indicate mutation. **(d)** Western blotting of Rlf in embryo head lysates from 14.5 dpc *Rlf*^*+/+*^, *RlfMomme*^*D8/D8*^, *Rlf*^*D28/D28*^ and *Rlf*^*D34/D34*^ revealed greatly reduced amounts of Rlf protein. Each track represents a different animal. Rlf protein was detected at approximately 280 kDa. Tubulin was used as a loading control. **(e**) Total, cytoplasmic and nuclear fractions of HeLa cells were isolated and protein concentration quantified. Equal amounts of each fraction were immunoblotted with anti-Rlf, GAPDH (cytoplasmic marker) and SMARCA5 (nuclear marker) antibodies, revealing nuclear localization of RLF. Rlf protein was detected at approximately 280 kDa, GAPDH protein at 37 kDa and SMARCA5 at approximately 120 kDa. **(f)** Coat color of offspring carrying the *A*^*vy*^ allele produced from a *Rlf*^*MommeD8*^ heterozygous female crossed to a pseudoagouti *A*^*vy*^ male. *Rlf*^*MommeD8*^ heterozygous offspring showed a shift in coat color towards pseudoagouti compared to wild-type littermates.

*MommeD30* was classified as an enhancer of variegation. We mapped the *MommeD30* mutation to a 1.9 Mbp interval on chromosome 17 encompassing 48 genes (Additional file 3). Exome deep sequencing identified a single base deletion in exon 5 of the *Wiz* gene, causing a frame shift mutation that is predicted to introduce a premature stop codon (Figure 2a and Table 1). No other putative ENU variants were identified in the linked region.

Wiz contains six Kruppel-type zinc finger motifs in a widely interspaced manner and has been reported to associate with the histone H3 lysine 9 (H3K9) methyltransferases G9a and GLP in cell lines [32,33]. No mouse mutants for *Wiz* have so far been described. Western blot analysis using an anti-Wiz antibody detected two bands (about 120 and 130 kDa), as expected, in wild-type embryos (Figure 2a). Embryos heterozygous for the *MommeD30* mutation showed approximately half the amount of Wiz protein and no Wiz protein was detected in homozygotes (Figure 2a). We conclude that *MommeD30* mice are haploinsufficient for Wiz. We refer to this allele as *Wiz*^*MommeD30*^.

### *MommeD18* carries a mutation in *Rap interacting factor 1* (*Rif1*)

*MommeD18* was classified as a suppressor of variegation and candidate gene sequencing revealed that *MommeD18* mice carry a nonsense mutation in exon 29 of the *Rif1* gene (Figure 2b and Table 1). Two other candidate genes (*Epc2* and *Mbd5*) were sequenced and no mutations were identified in these genes. Western blot analysis showed approximately half the amount of full-length Rif1 protein in *MommeD18* heterozygotes and no full-length Rif1 protein in a homozygote (Figure 2b). Furthermore, a *Rif1* gene trap allele (Materials and methods) had a similar effect on transgene expression as that observed with the *MommeD18* mutation, increasing the percentage of expressing cells in mice heterozygous for the gene-trap allele (Additional file 4).

We conclude that the *MommeD18* allele is the result of a mutation in *Rif1* that we refer to as *Rif1*^*MommeD18*^. *Rif1* has recently been shown to be involved in DNA double strand break repair [34-37] and replication timing in mammals [38,39], both processes involving significant chromatin reorganization. Our findings suggest a role for *Rif1* in transcription.

### *MommeD8*, *MommeD28* and *MommeD34* carry mutations in *Rearranged L-myc fusion* (*Rlf*), a gene about which little is known

Three enhancers of variegation, *MommeD8*, *MommeD28* and *MommeD34*, were found to carry mutations in *Rlf*. Whole interval capture revealed that *MommeD8* and *MommeD34* mice have mutations in exon 8 and exon 7, respectively. *MommeD8* is a missense mutation and *MommeD34* is a nonsense mutation (Figure 2c and Table 1). The *MommeD28* mutation was identified by candidate sequencing and affects the splice acceptor site of intron 4 (Figure 2c and Table 1). Western blot analysis using two independent anti-Rlf antibodies (one polyclonal and one monoclonal) detected an approximately 280 kDa band in protein lysates made from wild-type embryos (Figure 2d). Although the band detected on the western blots is substantially higher than the predicted molecular weight of 218 kDa (NCBI Mouse Build 37), this could be due to post-translational modifications to the Rlf protein. We concluded that it is likely that this band is Rlf. Reduced amounts (*MommeD8*) or no (*MommeD28* and *MommeD34*) Rlf protein was detected in homozygous embryos (Figure 2d). Together, these results suggest that *MommeD8* is a hypomorphic allele and that *MommeD28* and *MommeD34* are null alleles.

*Rlf* encodes a protein predicted to contain 16 widely spaced zinc finger domains, which has led to the suggestion that it has a role in transcriptional regulation [40]. Rlf is conserved across vertebrates. To determine the localization of Rlf in the cell, we carried out cell fractionation experiments. Rlf protein was specifically detected in the nuclear fraction of HeLa cells (Figure 2e). To our knowledge, we have identified the first mouse mutants for *Rlf* and hereafter refer to these alleles as *Rlf*^*MommeD8*^, *Rlf*^*MommeD28*^ and *Rlf*^*MommeD34*^.

We have identified *Rlf* from a screen that is based on the expression of a multi-copy variegating transgene. To test if *Rlf* is required for the establishment of epigenetic state at an endogenous locus, we studied the effect of *Rlf*^*MommeD8*^ on expression of *agouti viable yellow* (*A*^*vy*^). *A*^*vy*^ is a single copy gene that, like the transgene, is known to be sensitive to epigenetic state [41]. The *A*^*vy*^ allele is the result of an intracisternal-A-particle (IAP) retrotransposon insertion upstream of the agouti gene and isogenic mice carrying the *A*^*vy*^ allele can be yellow, mottled or pseudoagouti (dark brown). Coat color has been shown to correlate with the level of DNA methylation at the long-terminal repeat (LTR) promoter [42]. It has been shown that haploinsufficiency for modifiers of epigenetic reprogramming can alter the ratio of the different coat colors [5,6,43,44]. We crossed females heterozygous for *Rlf*^*MommeD8*^ with pseudoagouti *A*^*vy*^*/a* mice and scored the offspring for coat color. Offspring that inherited the *Rlf*^*MommeD8*^ allele were more likely to be pseudoagouti than their wild-type littermates, that is, haploinsufficiency for Rlf increased the probability of silencing at the *A*^*vy*^ locus (Figure 2f). This is consistent with *Rlf* being an enhancer of variegation and suggests that *Rlf* has a general role in epigenetic regulation.

### Novel alleles of known epigenetic regulators

**Figure 3:**
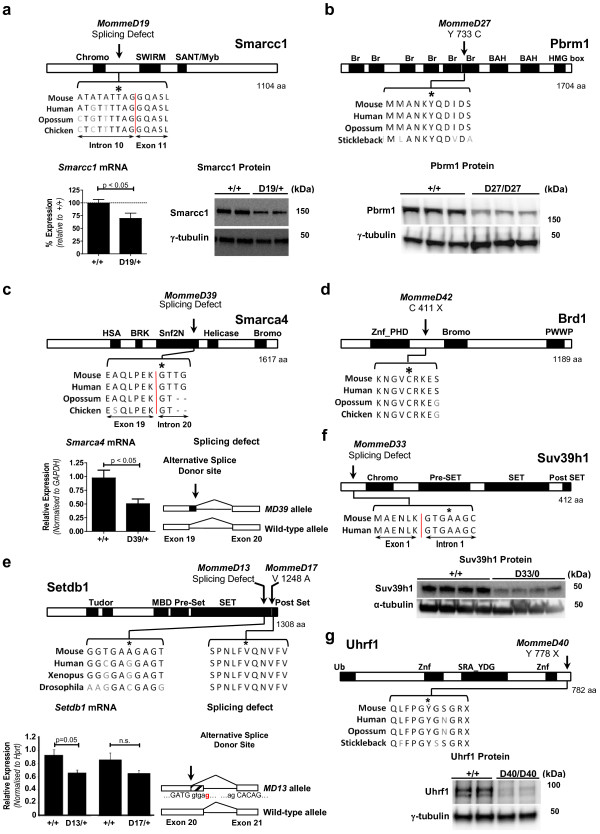
**Causative mutations in *MommeD19*, *MommeD27*, *MommeD39*, *MommeD42*, *MommeD13*, *MommeD33 *and *MommeD40*. (a)***MommeD19* carries a mutation at a splice site of *Smarcc1*. Real-time RT-PCR and immunoblotting of embryos at 12.5 days post-coitum (dpc) showed reduced levels of *Smarcc1* mRNA and Smarcc1 protein in heterozygotes (n ≥ 5 mice). Each track represents a different animal. Smarcc1 protein was detected at approximately 160 kDa. **(b)***MommeD27* harbors a mutation in a bromodomain of Pbrm1. Western blot analysis of 14.5 dpc embryo heads showed reduced levels of Pbrm1 in homozygotes. Each track represents a different animal. Pbrm1 was detected at approximately 190 kDa. **(c)***MommeD39* carries a mutation at a splice site of *Smarca4*. Real-time RT-PCR showed reduced *Smarca4* mRNA in testes of heterozygotes (n ≥ 4 mice). cDNA sequencing revealed that the mutation in *Smarca4*^*MommeD39*^ results in use of an alternative splice donor site. **(d)** The mutation in *MommeD42* introduces a premature stop codon at amino acid 411 of the Brd1 protein. **(e)** Mutations in *MommeD13* and *MommeD17* occur in the conserved SET domain of Setdb1. Real-time RT-PCR of *Setdb1* mRNA from testes of heterozygotes and age-matched wild types (n = 4 mice). cDNA analysis revealed that the *Setdb1*^*MommeD13*^ allele is associated with the use of an alternative splice donor site in exon 20, leading to a 62 bp truncation. **(f)***MommeD33* carries a mutation at a splice site in *Suv39h1*. Western blot analysis of Suv39h1 in adult thymus showed reduced Suv39h1 in hemizygous mutant males. Each track represents a different animal. Suv39h1 protein was detected at 48 kDa. **(g)** The mutation in *MommeD40* introduces a premature stop codon at amino acid 778 of Uhrf1. Western blot analysis of Uhrf1 revealed greatly reduced levels in 9.5 dpc embryos homozygous for the *Uhrf1*^*MommeD40*^ mutation. Each track represents a different animal. Uhrf1 protein was detected at approximately 90 kDa. Error bars indicate ± standard error of the mean. N.s., not significant. Asterisks indicate mutation.

In addition to the genes described above, we have produced 15 *MommeD* lines that carry mutations in genes already known to be involved in epigenetic regulation in the mouse. Exome deep sequencing of three suppressors of variegation, *MommeD19*, *MommeD27* and *MommeD39*, identified mutations in the chromatin remodelers *SWI/SNF related*, *matrix associated*, *actin dependent regulator of chromatin*, *subfamily c*, *member 1* (*Smarcc1*), *Polybromo 1* (*Pbrm1*) and *SWI/SNF related*, *matrix associated*, *actin dependent regulator of chromatin*, *subfamily a*, *member 4* (*Smarca4*), respectively (Table 1). *Smarcc1*^*MommeD19*^ carries a mutation in the splice acceptor site of intron 10 predicted to lead to an in-frame premature stop codon and likely nonsense-mediated mRNA decay. Quantitative real-time RT-PCR and western blot analysis showed that *Smarcc1* mRNA and Smarcc1 protein levels were reduced in heterozygous animals (Figure 3a). The *Pbrm1*^*MommeD27*^ mutation is a missense mutation in exon 17. Western blot analysis revealed reduced levels of Pbrm1 protein in homozygotes (Figure 3b). *Smarca4*^*MommeD39*^ has a mutation in the splice donor site of intron 20. cDNA sequencing revealed an extended exonic sequence (data not shown) that is predicted to lead to an in-frame premature stop codon and nonsense-mediated mRNA decay. Quantitative real-time RT-PCR analysis showed reduced *Smarca4* mRNA in heterozygotes (Figure 3c).

*MommeD42* was identified as an enhancer of variegation and carries a nonsense mutation in exon 11 of *Bromodomain containing 1* (*Brd1*; Figure 3d and Table 1). Brd1 has recently been reported to form a complex with the histone acetyltransferase HBO1 and is required for the transcriptional activation of the erythroid-specific regulator genes in fetal liver [45].

Three suppressors of variegation were found to carry mutations in H3K9 methyltransferases. *MommeD13* and *MommeD17* carry mutations in *Setdb1* and *MommeD33* has a mutation in *Suv39h1* (Table 1). The *Setdb1*^*MommeD13*^ mutation introduces a novel splice donor site leading to an in-frame premature stop codon in exon 20 (Figure 3e). cDNA analysis confirmed the presence of the predicted transcript (data not shown). *Setdb1*^*MommeD17*^ is a missense mutation in exon 21 of *Setdb1* (Figure 3e). RNA analysis indicates reduced levels of *Setdb1* mRNA in the *MommeD13* mutants whereas *Setdb1* mRNA levels were not significantly different in the *MommeD17* mutants (Figure 3e). The *Suv39h1*^*MommeD33*^ mutation is at the splice donor side of intron 20. This mutation is predicted to lead to a premature stop codon. Consistent with this, western blot analysis showed that the level of Suv39h1 protein was greatly reduced in hemizygous mutant males (*Suv39h1* is on the X chromosome; Figure 3f).

*MommeD40*, a suppressor of variegation, carries a nonsense mutation in exon 17 of the *Uhrf1* gene (Figure 3g and Table 1). Western blot analysis revealed greatly reduced levels of Uhrf1 protein in homozygous embryos (Figure 3g). Uhrf1 is known to associate with the maintenance DNA methyltransferase Dnmt1 [46] and has recently been shown to link DNA methylation and H3K9 methylation in human cells [47].

Seven *MommeD* lines are new mutant alleles of genes already identified in the screen and we have designated these lines *Baz1b*^*MommeD16*^, *Smchd1*^*MommeD23*^, *Trim28*^*MommeD31*^, *Dnmt1*^*MommeD32*^, *Smarca5*^*MommeD35*^, *Smchd1*^*MommeD36*^ and *Smarca5*^*MommeD37*^ (Table 1; Additional file 5).

**Figure 4:**
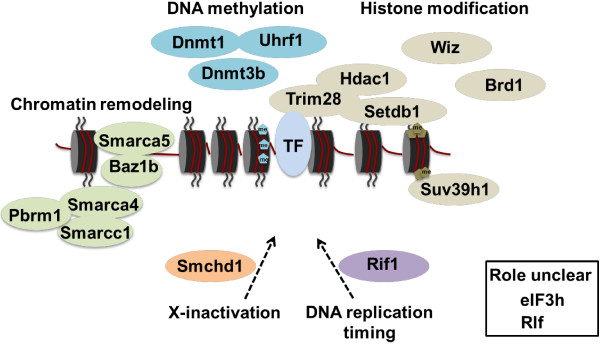
***MommeD *genes involved in transgene silencing in the mouse.** Using an ENU mutagenesis screen, 18 unique genes were identified to be involved in transgene silencing in the mouse. Factors are grouped by their broad mechanistic role in epigenetic regulation. TF, transcription factor; Me, methyl. Figure adapted from [2].

Approximately 60 Suppressor of variegation (*Su(var)*) and 25 Enhancer of variegation (*E(var)*) genes were identified in the *Drosophila* screens [2]. We have now documented 12 *Su(var)* and 8 *E(var)* genes in the mouse and these encode some of the key factors for DNA methylation and H3K9 methylation, as well as a large group of chromatin remodelers (Figure 4). Some of these genes, such as *Dnmt1*, *Suv39h* and *Trim28*, have been identified as core heterochromatin factors, whereas others, such as *Smarca5* and *Hdac1*, have been associated with gene silencing at euchromatic positions [2]. This is consistent with the variegating phenotype of the transgene. Interestingly, we have not yet recovered any members of the RNA interference/piwi-interacting RNA (piRNA) pathway or Polycomb and trithorax groups of proteins but the screen has not reached saturation.

### *Momme* genes are required for normal embryonic development

In most of the *MommeD* lines, heterozygous mice were observed at expected ratios at weaning and did not show any obvious phenotypes. To determine the viability of homozygotes, we performed embryonic dissections following intercrosses. We have focused on those lines carrying mutations in genes about which there is no or few data available in the literature.

**Figure 5:**
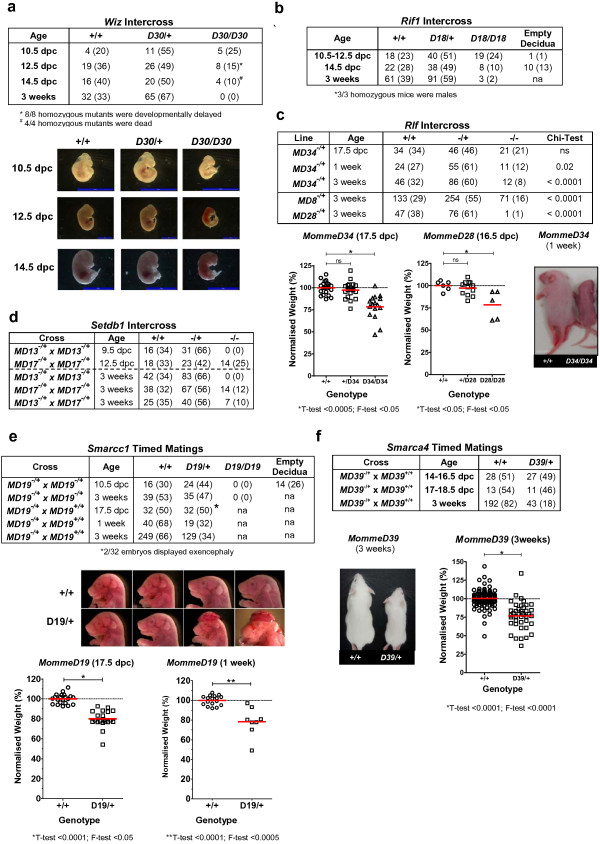
***MommeD *mutants show abnormal embryonic development.** Timed matings and intercrosses of *MommeD* mutants. **(a)***Wiz*^*MommeD30*^ mice; data show the number of mice observed (and in brackets the percentage) at 10.5 days post-coitum (dpc), 12.5 dpc, 14.5 dpc and at weaning. Representative embryos are shown. **(b)***Rif1*^*MommeD18*^ mice; data show the number of mice observed (and in brackets the percentage) at 10.5 to 12.5 dpc, 14.5 dpc and at weaning. na, not applicable. **(c)***Rlf* mutants; data show the number of mice observed (and in brackets the percentage). Embryonic weights were measured from intercrosses of *Rlf*^*MommeD34*^ and *Rlf*^*MommeD28*^ mice. Homozygous embryos from both had a significant increase in weight variation at 17.5 dpc or 16.5 dpc, respectively. Weights for each litter were normalized to the average weight of wild-type embryos in that litter. Each data point represents an individual. Homozygotes were smaller than wild-type littermates at one week after birth. ns, not significant. **(d)***Setdb1*^*MommeD13*^ and *Setdb1*^*MommeD17*^ mice; data show the number of mice observed (and in brackets the percentage). **(e)***Smarcc1*^*MommeD19*^ and *Smarcc1*^*MommeD19*^ mated to wild-type mice revealed incomplete penetrance of heterozygous lethality after birth. Data show the number of mice (and in brackets the percentage). Weights of 17.5 dpc *Smarcc1*^*MommeD19*^ embryos and pups were, on average, less than that of wild-type littermates (T-test, *P* < 0.0001) and showed greater variation (F-test, *P* < 0.05). Weights in each litter were normalized to the average weight of wild-type embryos in that litter. Each data point represents an individual. **(f)***Smarca4*^*MommeD39*^ heterozygotes showed reduced viability. Data show the number of mice and in brackets the percentage. Heterozygotes were smaller than their wild-type littermates. *Smarc4*^*MommeD39/+*^ weighed less (T-test, *P* < 0.0001) and weights were more variable (F-test, P<0.0001) than wild types. Weights in each litter were normalized to the average weight of wild-type embryos in that litter. Each data point represents an individual.

Following *Wiz*^*MommeD30*^ intercrosses, no homozygous animals were recovered at weaning out of 97 progeny (Figure 5a). To determine when *Wiz*^*MommeD30*^ homozygotes died, embryos from intercross matings were obtained at different stages of development and genotyped. Viable homozygotes were recovered at the expected ratios at 10.5 days post-coitum (dpc). However, these embryos appeared smaller than their wild-type and heterozygous littermates. At 12.5 dpc homozygotes were present in numbers less than expected (15% as opposed to 25%) and at 14.5 dpc no viable homozygous embryos were obtained (Figure 5a). We conclude from these data that embryonic death in mice homozygous for *Wiz*^*MommeD30*^ occurs between 10.5 and 12.5 dpc.

*Rif1*^*MommeD18*^ intercrosses produced wild-type and heterozygous offspring at the expected ratios and occasional homozygous individuals (3 out of 155 offspring) at weaning (Figure 5b). All surviving homozygotes were males and two, out of three tested, were infertile. Timed matings revealed that the majority of the homozygotes died during the second half of gestation (Figure 5b). This is consistent with the literature [48].

*Rlf* intercrosses produced homozygous individuals that survived to weaning in less than expected numbers for *Rlf*^*MommeD8*^, *Rlf*^*MommeD28*^ and *Rlf*^*MommeD34*^ (Figure 5c). *Rlf*^*MommeD34*^ homozygotes died in the first week after birth, for unknown reasons (Figure 5c). A similar finding was made for *Rlf*^*MommeD28*^ homozygotes; most died in the first week after birth (data not shown). However, in both lines some homozygotes did survive to adulthood and were fertile (data not shown). Embryonic dissections indicated that, for the *Rlf*^*MommeD28*^ and *Rlf*^*MommeD34*^ lines, late gestation homozygous embryos weighed significantly less than their heterozygous or wild-type littermates (T-test, *P* < 0.05; Figure 5c). *Rlf*^*MommeD8*^ behaves like a hypomorphic allele, with approximately half the expected number of homozygotes present at weaning (Figure 5c). Those surviving were smaller and were fertile (data not shown).

One advantage of ENU mutagenesis is that it produces point mutations, increasing the likelihood of hypomorphic mutations. We have reported hypomorphic alleles for *Smarca5*^*MommeD4*^ and *Dnmt3b*^*MommeD14*^ previously [6,10]. In addition to the *Rlf*^*MommeD8*^ allele described above, *Setdb*^*MommeD17*^ behaves like a hypomorphic allele for the following reasons. Following intercrosses, some *Setdb1*^*MommeD17*^ homozygotes were present at weaning (Figure 5d) and some survived to adulthood but were smaller than their wild-type and heterozygous littermates and showed decreased fertility (data not shown). Mice homozygous for a null allele of *Setdb1* have been reported to die at the early post-implantation stage [49]. Consistent with this, timed matings with *Setdb1*^*MommeD13*^ heterozygous mice produced no viable homozygous offspring at 9.5 dpc (Figure 5d). To our knowledge, *Setdb1*^*MommeD17*^ is the first hypomorphic allele for *Setdb1*.

In two cases, *Smarcc1*^*MommeD19*^ and *Smarca4*^*MommeD39*^, heterozygous mutants showed signs of stochastic death prior to weaning. Following *Smarcc1*^*MommeD19*^ intercrosses, no viable homozygotes were obtained at weaning and timed matings revealed that homozygous lethality occurred prior to mid-gestation, as expected based on the literature [50] (Figure 5e). At weaning the percentage of heterozygotes to wild types (47% and 53%, respectively) was less than that expected (66% and 33%, respectively). Maintenance of the colony, which involved heterozygous to wild type crosses, also produced fewer mutants than expected at weaning (34% as opposed to 50%) (Figure 5e). Further investigation suggested that this stochastic death of heterozygotes was occurring after 17.5 dpc and before 1 week postnatal (Figure 5e). At 17.5 dpc, some heterozygotes had exencephaly, although this could not account for all the perinatal lethality observed (Figure 5e). The average weight of heterozygous mice at E17.5 and at 1 week postnatal was reduced (T-test, *P* < 0.0001) and greater variance (F-test, *P* < 0.05) was observed among heterozygotes than among wild-type littermates (Figure 5e).

Maintenance of the *Smarca4*^*MommeD39*^ colony (heterozygote to wild type crosses) also revealed non-Mendelian ratios; heterozygotes were present in less than expected numbers at weaning (18% as opposed to 50%) and were smaller than their wild-type littermates (Figure 5f). Timed matings showed that the ratios were close to Mendelian prior to birth (Figure 5f). Body weights of heterozygotes were lower than those of wild-type littermates at weaning (T-test, *P* < 0.0001) and, again, the variance was greater in the heterozygotes (F-test, *P* < 0.0001) (Figure 5f). Similar stochastic death of heterozygotes has been described by others for mice with knock-out alleles for *Smarcc1* and *Smarca4*[50,51]. In these cases, however, the genetic heterogeneity among the mice could have been an underlying factor.

We have previously reported increased variance in body weight and behavioral responses in adult mice heterozygous for *Trim28*[8] and others have made similar findings with respect to *Trim28* during the oocyte to embryo transition [52]. We have also reported that reduced levels of Dnmt3a are associated with an increase in body weight variance in adults [8]. These observations, in the context of inbred strains reared in controlled environments, are consistent with a role for the genes identified in this screen in canalization, a term coined by Waddington to describe phenotypic robustness during development. Exactly how important probabilistic developmental events are in determining phenotype is not yet clear [53]. We have so far reported only three measures; the penetrance of homozygous or heterozygous death in the colony, the body weight of heterozygotes or homozygotes (at various ages) in the colony and behavioral responses. More extensive phenotyping of these inbred *MommeD* lines should enable us to gain a better understanding of this phenomenon.

In the remaining *MommeD* lines examined, *Baz1b*^*MommeD16*^, *Smchd1*^*MommeD23*^, *Pbrm1*^*MommeD27*^, *Trim28*^*MommeD31*^, *Dnmt1*^*MommeD32*^, *Suv39h1*^*MommeD33*^, *Smarca5*^*MommeD35*^, *Smchd1*^*MommeD36*^, *Smarca5*^*MommeD37*^, *Uhrf1*^*MommeD40*^ and *Brd1*^*MommeD41*^, we observed homozygous embryonic death that is similar to that reported for knock-out alleles of these genes (Additional file 6).

### DNA methylation levels at the transgene are increased in *Rlf* mutants

**Figure 6:**
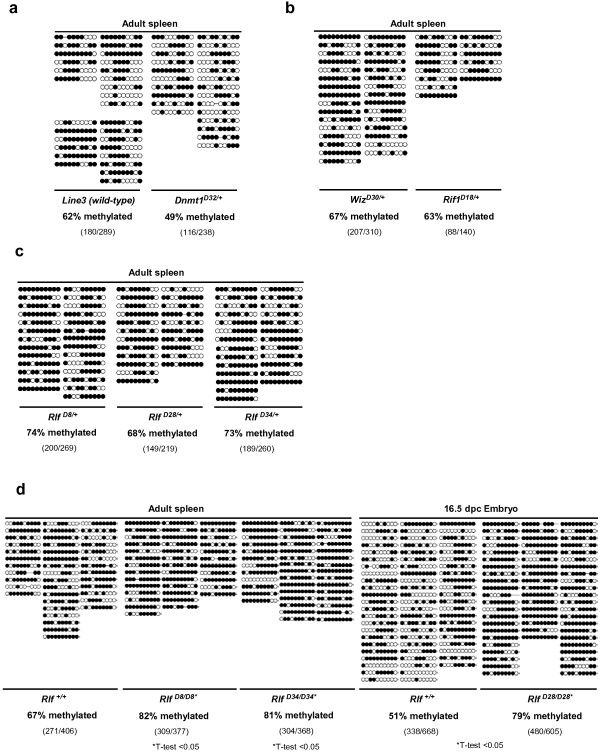
**Bisulfite sequencing of the HS-40 enhancer region of the GFP transgene. (a)** DNA methylation levels were reduced in *Dnmt1*^*MommeD32*^ heterozygous mice compared to wild-type mice in adult spleens (n = 4 wild-type mice and n = 2 *Dnmt1*^*MommeD32*^ mutant mice). Filled circles represent methylated CpG sites. **(b)** DNA methylation levels in adult spleens were unaffected in mice heterozygous for *Wiz*^*MommeD30*^ and *Rif*^*MommeD18*^ (n = 4 for *Wiz*^*MommeD30*^ and n = 2 for *Rif1*^*MommeD18*^). **(c)** DNA methylation levels in adult spleens of *Rlf*^*MommeD8*^, *Rlf*^*MommeD28*^ and *Rlf*^*MommeD34*^ heterozygotes were increased compared to wild-type mice (n = 2 mice for all genotypes). **(d)***Rlf* homozygotes showed significantly increased DNA methylation (T-test, *P* < 0.05) in adult male spleen (*Rlf*^*MommeD8*^ and *Rlf*^*MommeD34*^) and 16.5 dpc embryo (*Rlf*^*MommeD28*^) compared to wild types (n = 3 for all genotypes).

Changes in transcription can correlate with changes in DNA methylation at transgenes and metastable epialleles [42,54-56]. We have shown previously that changes in the percentage of red blood cells expressing GFP can be accompanied by changes in DNA methylation at the transgene [7,10]. Using bisulfite sequencing, we investigated DNA methylation at the HS-40 enhancer region of the transgene in *MommeD* lines. We used adult spleens from wild-type mice and mice heterozygous for a *MommeD* mutation. Consistent with our previous reports, in *Line3* wild-type mice around 60% of the CpGs in the HS-40 element were methylated (Figure 6a). Mice heterozygous for *Dnmt1*^*MommeD32*^ suggested a decrease in CpG methylation (around 50%; Figure 6a). This is consistent with an increase in expression of the GFP transgene in these mutants and with its role as the maintenance DNA methyltransferase. In mice heterozygous for the *Wiz* and *Rif1* mutations DNA methylation patterns at the HS-40 region were unaffected (Figure 6b).

In the case of the *Rlf* alleles, we detected higher levels of DNA methylation at the HS-40 element in adult spleens in *Rlf*^*MommeD8*^ (82%, T-test <0.05) and *Rlf*^*MommeD34*^ (81%, T-test <0.05) homozygotes compared to wild types (67%) (Figure 6d). A similar trend (not statistically significant) was observed in the *Rlf*^*MommeD8*^ and the *Rlf*^*MommeD34*^ heterozygotes compared with that seen in *Line3* wild-type (Figure 6a,c). In the case of *Rlf*^*MommeD28*^, where adult homozygotes are extremely rare, we analyzed DNA methylation patterns in 16.5 dpc embryos and made a similar finding. The HS-40 element was more methylated in *Rlf*^*MommeD28*^ homozygotes (79%, T-test <0.05) than it was in wild-type controls (51%) (Figure 6d). This is the first time that we have observed hypermethylation of the transgene in a *MommeD* line. An increase in DNA methylation at the transgene locus in the *Rlf* mutants is consistent with the decreased expression of the transgene in these mice.

## Conclusions

Our results suggest that transgene silencing in the mouse acts through a mechanism common to transposon silencing, X-inactivation and imprinting. We provide evidence that a novel gene, *Rlf*, is involved in this process. Depletion of *Rlf* leads to DNA hypermethylation at the transgene. How this is achieved is currently unknown and requires further investigation. We have isolated the first mouse mutants for *Rlf* and *Wiz* and shown that the genes are required for normal embryonic development. Notably, the human homologs of many of the genes recovered from our screen have been found to be associated with human diseases, in some cases identified in family studies and in other cases suggested by genome-wide association studies (Table 1).

Since the mouse is often used as a disease model, we anticipate that our collection of *Momme* mutants will provide a valuable resource for researchers across many disciplines.

## Material and methods

### Generation and screening of mutant mice

Procedures were approved by the Animal Ethics Committee of the QIMR Berghofer Medical Research Institute. The ENU screen was carried out in the FVB/NJ inbred transgenic line, *Line3*, which is homozygous for a multicopy GFP transgene, as described previously [5]. All *MommeD* lines were maintained in this background and are homozygous for the GFP transgene. All experimental data: crosses to other lines, intercrosses, and so on, were carried out using heterozygous *MommeD* mice five generations or more removed from the *MommeD* founder.

### Other mouse strains

The congenic strain, *Line3C*, used for linkage studies, was produced by crossing *Line3* to C57BL/6J for 10 generations, selecting for mice carrying the transgene by flow cytometry. Inbred C57BL/6J mice were purchased from ARC Perth (Perth, WA, Australia). *Rif1*^*GT*^ mice were generated by the Australian Phenomics Network from an embryonic stem cell clone carrying a trapped Rif1 allele (A045A01; German Gene Trap Consortium), crossed to *Line3C* (C57BL/6J) and maintained on this background.

### Flow cytometry

Mice were analyzed by flow cytometry at 3 weeks of age. A drop of blood was collected in Osmosol buffer and analyzed on a Guava easyCyte HT (Merck/Millipore, Darmstadt, Germany). The data were analyzed by using Guava InCyte software with a GFP-positive gate set to exclude 99.9% of wild-type erythrocytes. Histograms shown depict only the GFP fluorescence channel.

### Linkage analysis

Heterozygous mutant *MommeD* mice, at least four generations down from the founder, were backcrossed twice to *Line3C* (see above) and phenotyped for GFP expression by flow cytometry. DNA from tail tips was used to perform linkage analysis. The Illumina GoldenGate genotyping assay (Mouse Medium Density Linkage Panel) was used for *MommeD13*, *MommeD16* to *MommeD19*, *MommeD27*, *MommeD30*, *MommeD32*, *MommeD34*, *MommeD35*, *MommeD37*, *MommeD39*, *MommeD40* and *MommeD42* on at least 12 wild-type and 12 heterozygous mice from each line. The Mouse Medium Density Linkage panel contains 766 measurable SNPs between C57BL/6J and FVB/NJ. Samples were genotyped following the Illumina protocol and genotype calls were made using the Genotyping module of the GenomeStudio v1.1 software. Only samples with a call rate >95 were accepted. Linked intervals were identified based on LOD scores of 3 or higher (Additional file 7).

For *MommeD8*, *MommeD23*, *MommeD28*, *MommeD32*, *MommeD33* and *MommeD36* the linked chromosomes were identified using microsatellite or SNP markers. Additional markers were used to reduce the linked intervals using wild-type and heterozygous mice. Mice wild-type for the mutation should only have C57BL/6J chromosomes in the linked region, while mice heterozygous for the mutation should carry both C57BL/6J and FVB/NJ chromosomes.

For *MommeD13*, *MommeD16*, *MommeD18*, *MommeD23*, *MommeD28*, *MommeD32*, *MommeD33* and *MommeD35*, mutations were found using Sanger sequencing. For the remaining *MommeD* lines deep sequencing approaches were used.

### Whole exome deep sequencing and interval capture

Mouse exomes were captured and sequenced using exome capture reagents from either Agilent (SureselectXT Mouse All Exon v.1, Santa Clara, CA, USA) or Roche NimbleGen (SeqCap EZ Mouse Exome, version Beta 2, 110603_MM9_exome_rebal_2EZ_HX1, Madison, WI, USA). The Agilent captures were carried out as detailed in the manufacturer’s protocol (version 1.1.1), with the following exceptions: a Bioruptor (Diagenode, Liège, Belgium) was used for DNA shearing, producing fragment sizes of approximately 200 to 300 bp, and sample pooling/multiplexing was omitted. The Bioruptor sonication settings were 3 × 10 minutes on the low setting with cycles of 30 seconds on and 30 seconds off. The Roche NimbleGen captures were carried out as outlined in the Illumina optimized Roche NimbleGen SeqCap User’s Guide (version 1.0), also using the Bioruptor for DNA fragmentation.

For two mutant lines, *MommeD8* and *MommeD34*, a 4.2 Mbp linked interval was sequenced in full using a custom capture array designed and manufactured by Roche NimbleGen. *MommeD8* was captured and sequenced by Roche NimbleGen using Roche 454 sequencing (110317_MM9_mommeD8_Rocky_cap_HX3). Three mice were sequenced; a wild type, a heterozygote and a homozygote. For *MommeD34*, a heterozygous mutant was captured and sequenced in-house using the same array capture design on 2.1M arrays with HX3 mixers, according to the Roche NimbleGen Arrays User’s Guide, Illumina Optimized protocol (version 1.0).

### SNP calling

The sequencing reads from the targeted sequence capture experiments were aligned to the mouse genome (build 37, mm9) using the program bwa version 0.6.1 [57]. Datasets generated by Roche on the 454 platform were mapped using bwa bwasw [58] while the remaining datasets, generated on the Illumina HiSeq or GAIIx platforms, were mapped using bwa aln, with default settings, followed by bwa sampe with the default settings. The resulting sam files were converted to bam files and coordinate-sorted using SAMtools version 0.1.17 [59] and PCR duplicates were subsequently eliminated using the program Picard MarkDuplicates, version 1.48 [60].

For each sample, nucleotide variants were identified within the intervals for which linkage had previously been determined. This was achieved by creating a pileup file of the linked region using SAMtools mpileup, using the option -q 20, followed by variant calling using the program Varscan version v2.2.8 [61] using the 'somatic' feature and the settings -min-coverage 15 and -min-var-freq 0.3. Varscan somatic calls sequence differences between a case and a control sample; as control the sequenced exome from a different ENU mutant was used. The case and control exomes were constructed using the same library preparation methods and sequenced in the same deep sequencing run, but had different linked intervals. All exomes were mapped and processed in parallel, using identical settings, to minimize post-sequencing artifacts. The output from Varscan was manually screened for likely ENU mutations, appearing as heterozygous SNPs in the mutant and wild type in the control. These SNPs were in turn validated using the Sanger method.

The custom capture and sequencing of the *MommeD34* linked region was carried out in-house without a matched wild-type control; instead, a merge of the three *MommeD8* deep sequencing samples previously sequenced by Roche was used as control. A merge of these 454 datasets was used in order to achieve greater read depth across the region. SNP calling was subsequently carried out as described above. Exome sequencing datasets generated in this study are accessible via European Nucleotide Archive (ENA) under accession ERP003831.

### Genotyping

Once the mutations had been identified, genotyping was carried out by either sequencing (*MommeD13*, *MommeD16*, *MommeD17*, *MommeD18*, *MommeD23*, *MommeD27*, *MommeD28*, *MommeD30*, *MommeD32*, *MommeD34*, *MommeD35*, *MommeD36*, *MommeD37*, *MommeD39*, *MommeD40* and *MommeD42*) or, if the mutation had changed a restriction enzyme recognition site, by PCR and digestion and gel electrophoresis (*MommeD8* and *MommeD19*). Genotyping primers are provided in Additional file 8 and Sanger sequencing traces in Additional file 9.

### Embryo dissections

All embryos were produced by natural matings and detection of a vaginal plug was counted as 0.5 dpc. Except where otherwise stated, embryos were produced by intercrosses.

### Introduction of the mutant lines into mice carrying the *A*^*vy*^ allele

FVB/NJ mice heterozygous for the *MommeD8* mutation and homozygous for the GFP transgene were mated with C57BL/6J mice heterozygous for the *A*^*vy*^ allele. The coat color phenotype was classified at weaning by a trained observer as either yellow, mottled, or agouti. GFP expression was determined by flow cytometry and used to classify the mice into mutants or wild-type for the *MommeD8* mutation. FVB/NJ mice carry the *A* locus and C57BL/6J mice carry the *a* locus. Yellow and mottled offspring carry the *A*^*vy*^ allele. All agouti-colored offspring were genotyped by PCR, to assess whether they were *A*^*vy*^*/A* and pseudoagouti, or *A/a*, as reported [56].

### Bisulfite sequencing of the transgene HS-40 enhancer

Bisulfite conversion of DNA was carried out using the EpiTect Bisulfite Kit (Qiagen, Doncaster, VIC, Australia) according to the manufacturer’s instructions. At least two male adult spleens were used for each *MommeD* line. The bisulfite conversion rate was at least 97% and sequences were analyzed using the BiQ Analyser software [62]. Oligonucleotides to the bisulfite converted HS-40 enhancer region were as follows (5’-3’): GFPbisF1: AAAATAAAATTTTTGGATTGTTATTATTATAA; GFPbisF2: ATATTTGTAATTTTAGTATTTTGGGAGGTT and GFPbisR: AATCTCTACTCACTACAAACTCCATCTC. Cycling conditions were as follows: 94°C for 2 minutes for 1 cycle; 94°C for 30 seconds, 60°C for 30 seconds, 72°C for 45 seconds for 35 cycles and 72°C for 6 minutes for 1 cycle.

### Protein analysis

Whole-cell extracts were prepared from various tissues of adult mice. Protein concentration was measured by BCA (Thermo Scientific, Waltham, MA, USA) and protein lysates were separated using polyacrylamide gels (Invitrogen, Carlsbad, CA, USA, BioRad, Hercules, CA, USA or ECL, Rydalmere, NSW, Australia). Antibodies used were as follows: anti-γ-tubulin (T5192, Sigma-Aldrich, St. Louis, MO, USA), anti-Rif1 (ab13422, Abcam, Cambridge, ENG, UK), anti-Wiz (gift from Yoichi Shinkai), anti-Smarcc1 (9053S, Cell Signaling, Danvers, MA, USA), anti-Rlf (ab115011, Abcam - rabbit polyclonal) and (M05, clone2G2, Abnova - mouse monoclonal, Neihu District, Taipei City, Taiwan), anti-Pbrm1 (Rabbit polyclonal ABE70, Millipore), anti-Suv39h1 (clone D11B5, Cell Signaling), anti-Uhrf1 (ab151187 Abcam), anti-GAPDH (D16H11 XP, Cell Signaling) and anti-Snf2H (ab3749, Abcam).

### Cell fractionation

HeLa cells were trypsinized, pelleted and washed twice with phosphate-buffered saline before re-suspending in ice-cold cytoplasmic extraction buffer (10 mM Tris pH 7.4, 10 mM NaCl, 3 mM MgCl_2_, 0.1% NP-40). A small aliquot of this suspension, the total cell lysate, was transferred to a separated tube. The remaining suspension was then centrifuged (500 rpm, 10 minutes, 4°C) to pellet the nuclei. The supernatant (cytoplasmic fraction) was aliquoted into a separate tube. Nuclei were re-suspended in an 8 M urea lysis buffer (8 M urea, 1/10 vol. glycerol, 1/20 vol. 20% SDS, 1/2,000 vol. 1 M dithiothreitol, 1/100 vol. 1 M Tris, pH 6.8).

### RNA isolation, cDNA analysis and quantitative real-time RT-PCR

Total RNA was extracted from various tissues using TRI reagent (Invitrogen). cDNA was synthesized from total RNA using SuperScriptIII reverse transcriptase (Invitrogen) or AMV reverse transcriptase (Roche) and random hexamer primers. Quantitative real-time PCR was performed with the Platinum SYBR Green qPCR Super Mix -UDG (Invitrogen) with primers designed to span exon/intron boundaries. All reactions were performed in triplicates and normalized to Hprt or Gapdh. PCRs were run on a Viia7 (Applied Biosystems, Mulgrave, VIC, Australia) or on a Corbett Research Rotor-Gene (Qiagen). Cryp-Skip [63] was used for splice site prediction. cDNA from mutant alleles was sequenced using Sanger sequencing. Primer sequences are provided in Additional file 8.

### Statistical analysis

Statistical significance of quantitative data was determined by two-tailed Student’s T-test. F-test was used to test whether variance was significantly different between wild-type and mutant groups. The proportions of genotypes were compared to expected Mendelian ratios using a χ^2^ test. For all datasets a minimum of three biological replicates were analyzed.

## Abbreviations

bp: base pair; dpc: days post-coitum; ENU: N-ethyl-N-nitrosourea; FSHD2: facioscapulohumeral dystrophy type 2; GFP: green fluorescent protein; H3K9: histone H3 lysine 9; Momme: *Modifier of murine metastable epiallele*; PCR: polymerase chain reaction; SNP: single-nucleotide polymorphism.

## Competing interests

The authors declare no competing financial interests.

## Authors’ contributions

LD, SKH, and HO contributed to study design, carried out experiments, interpreted results and helped to draft the manuscript. TE, LI, EH, NW, AA, AS, JY, VB, JS, AA, ZP, NW, DG, MEB and JAJ carried out experiments and interpreted results. EW conceived the study and project design, performed ENU mutagenesis, interpreted results and helped to draft the manuscript. All authors read and approved the final manuscript.

## Supplementary Material

Additional file 1 **Linked intervals.** Manhattan plots showing linked intervals identified by Illumina GoldenGate SNP genotyping analysis. The x-axis represents the chromosomes and the y-axis is the LOD score. Peaks with a LOD score of 3 or higher are considered significant. Click here for file

Additional file 2 **Flow cytometric profiles of the *MommeDs*. (a)** Representative flow cytometry profiles show the percentage of GFP-expressing erythrocytes from wild-type and heterozygous mutant littermates (n = 3). The x-axis represents the erythrocyte fluorescence on a logarithmic scale and the y-axis is the number of cells detected at each fluorescence level. **(b)** Percentage of GFP-expressing cells in wild-type, heterozygous mutant and homozygous mutant (where viable) mice at three weeks of age (mean ± standard error of the mean). Click here for file

Additional file 3 **Mapping interval for *MommeD30. *(a)***MommeD30* was produced in the FVB/NJ strain of mice and mapped by crossing twice onto Line3C in a C57BL/6J background. The results of the genotype for seven SNP markers and one microsatellite marker surrounding the linked interval are shown. The number of mice classified into each haplotype is shown on top. Our estimate of the linked interval is between rs29539305 and rs33446195 on chromosome 17 (highlighted). **(b)** List of genes in the *MommeD30* linked interval on chromosome 17. The *Mus musculus* Ensembl database (release 37) was used to export a list of transcripts (protein coding and non-coding RNAs) within the 1.9 Mbp *MommeD30* interval. Click here for file

Additional file 4 **GFP expression in offspring of a *Rif1***^***GT***^**heterozygote crossed to *Line3C*.** A *Rif1* gene trap allele (*Rif1*^*GT*^) had a similar effect on transgene expression as that observed with the *MommeD18* mutation, increasing the percentage of expressing cells in mice heterozygous for the gene-trap allele. Click here for file

Additional file 5 **Novel mutant alleles of *Baz1b, Smchd1, Trim28, Dnmt1 *and *Smarca5*. (a)***MommeD16* carries a point mutation resulting in a non-conservative amino acid change in the Wstf domain of Baz1b. **(b)***MommeD23* and *MommeD36* carry point mutations in *Smchd1*. Both mutations introduce premature stop codons in the Smchd1 protein. **(c)***MommeD31* carries a point mutation in *Trim28* that results in an amino acid change in a highly conserved zinc finger domain. **(d)***MommeD32* carries a point mutation that results in an amino acid change in the BAH domain of Dnmt1. **(e)***MommeD35* and *MommeD37* carry mutations that result in amino acid changes in highly conserved domains of the Smarca5 protein. Click here for file

Additional file 6 **Embryonic development in *MommeD *mutants.** Tabulated data shows the number of observed mice and in brackets the percentage of total. **(a)** Intercrosses of *Pbrm1*^*MommeD27*^ mice produce no homozygous offspring at three weeks. **(b)***Brd1*^*MommeD42*^ homozygous mice are embryonic lethal around 10.5 dpc. **(c)** Mice carrying mutations in *Suv39h1*^*MommeD33*^ are viable at three weeks. **(d)***Uhrf1*^*MommeD40*^ homozygotes are embryonic lethal around 10.5 dpc. **(e)** Some homozygous *Baz1b*^*MommeD16*^ mice were obtained at three weeks but less than expected. **(f)** No (*Smchd1*^*MommeD36*^) or few (*Smchd1*^*MommeD23*^) homozygous individuals were recovered from intercrosses. The *Smchd1*^*MommeD23*^ homozygotes that survived were males. **(g)***Trim28*^*MommeD31*^ homozygous mice are embryonic lethal prior to 10.5 dpc. **(h)** Mice homozygous for the *Dnmt1*^*MommeD32*^ mutation die around 10.5 dpc. **(i)** Intercrosses of *Smarca5*^*MommeD35*^ and *Smarca5*^*MommeD37*^ produced no homozygous offspring at three weeks. Timed matings show that survival of homozygous mutants at 12.5 to 14.5 dpc was rare in *Smarca5*^*MommeD35*^ mutants. In *Smarca5*^*MommeD37*^ mutants homozygous embryonic death occurred prior to 14.5 dpc. Click here for file

Additional file 7 Linkage analysis using Illumina GoldenGate genotyping assay. Click here for file

Additional file 8 List of primer sequences. Click here for file

Additional file 9 Sanger traces. Click here for file

## References

[B1] Henikoff S Position-effect variegation after 60 years Trends Genet 1990 14 422 426 2087785 10.1016/0168-9525(90)90304-o

[B2] Fodor BD Shukeir N Reuter G Jenuwein T Mammalian Su(var) genes in chromatin control Annu Rev Cell Dev Biol 2010 14 471 501 10.1146/annurev.cellbio.042308.113225 19575672

[B3] Schotta G Ebert A Dorn R Reuter G Position-effect variegation and the genetic dissection of chromatin regulation in Drosophila Semin Cell Dev Biol 2003 14 67 75 10.1016/S1084-9521(02)00138-6 12524009

[B4] Reuter G Spierer P Position effect variegation and chromatin proteins Bioessays 1992 14 605 612 10.1002/bies.950140907 1365916

[B5] Blewitt ME Vickaryous NK Hemley SJ Ashe A Bruxner TJ Preis JI Arkell R Whitelaw E An N-ethyl-N-nitrosourea screen for genes involved in variegation in the mouse Proc Natl Acad Sci U S A 2005 14 7629 7634 10.1073/pnas.0409375102 15890782 PMC1140414

[B6] Chong S Vickaryous N Ashe A Zamudio N Youngson N Hemley S Stopka T Skoultchi A Matthews J Scott HS de Kretser D O'Bryan M Blewitt M Whitelaw E Modifiers of epigenetic reprogramming show paternal effects in the mouse Nat Genet 2007 14 614 622 10.1038/ng2031 17450140 PMC3199608

[B7] Ashe A Morgan DK Whitelaw NC Bruxner TJ Vickaryous NK Cox LL Butterfield NC Wicking C Blewitt ME Wilkins SJ Anderson GJ Cox TC Whitelaw E A genome-wide screen for modifiers of transgene variegation identifies genes with critical roles in development Genome Biol 2008 14 R182 10.1186/gb-2008-9-12-r182 19099580 PMC2646286

[B8] Whitelaw NC Chong S Morgan DK Nestor C Bruxner TJ Ashe A Lambley E Meehan R Whitelaw E Reduced levels of two modifiers of epigenetic gene silencing, Dnmt3a and Trim28, cause increased phenotypic noise Genome Biol 2010 14 R111 10.1186/gb-2010-11-11-r111 21092094 PMC3156950

[B9] Daxinger L Oey H Apedaile A Sutton J Ashe A Whitelaw E A forward genetic screen identifies eukaryotic translation initiation factor 3, subunit H (eIF3h), as an enhancer of variegation in the mouse G3 (Bethesda) 2012 14 1393 1396 2012 23173090 10.1534/g3.112.004036 PMC3484669

[B10] Youngson NA Epp T Roberts AR Daxinger L Ashe A Huang E Lester KL Harten SK Kay GF Cox T Matthews JM Chong S Whitelaw E No evidence for cumulative effects in a Dnmt3b hypomorph across multiple generations Mamm Genome 2013 14 206 217 10.1007/s00335-013-9451-5 23636699

[B11] Blewitt ME Gendrel AV Pang Z Sparrow DB Whitelaw N Craig JM Apedaile A Hilton DJ Dunwoodie SL Brockdorff N Kay GF Whitelaw E SmcHD1, containing a structural-maintenance-of-chromosomes hinge domain, has a critical role in X inactivation Nat Genet 2008 14 663 669 10.1038/ng.142 18425126

[B12] Gendrel AV Apedaile A Coker H Termanis A Zvetkova I Godwin J Tang YA Huntley D Montana G Taylor S Giannoulatou E Heard E Stancheva I Brockdorff N Smchd1-dependent and -independent pathways determine developmental dynamics of CpG island methylation on the inactive X chromosome Dev Cell 2012 14 265 279 10.1016/j.devcel.2012.06.011 22841499 PMC3437444

[B13] Leong HS Chen K Hu Y Lee S Corbin J Pakusch M Murphy JM Majewski IJ Smyth GK Alexander WS Hilton DJ Blewitt ME Epigenetic regulator Smchd1 functions as a tumor suppressor Cancer Res 2013 14 1591 1599 10.1158/0008-5472.CAN-12-3019 23269277

[B14] Lemmers RJ Tawil R Petek LM Balog J Block GJ Santen GW Amell AM van der Vliet PJ Almomani R Straasheijm KR Krom YD Klooster R Sun Y den Dunnen JT Helmer Q Donlin-Smith CM Padberg GW van Engelen BG de Greef JC Aartsma-Rus AM Frants RR de Visser M Desnuelle C Sacconi S Filippova GN Bakker B Bamshad MJ Tapscott SJ Miller DG van der Maarel SM Digenic inheritance of an SMCHD1 mutation and an FSHD-permissive D4Z4 allele causes facioscapulohumeral muscular dystrophy type 2 Nat Genet 2012 14 1370 1374 10.1038/ng.2454 23143600 PMC3671095

[B15] Klein CJ Botuyan MV Wu Y Ward CJ Nicholson GA Hammans S Hojo K Yamanishi H Karpf AR Wallace DC Simon M Lander C Boardman LA Cunningham JM Smith GE Litchy WJ Boes B Atkinson EJ Middha S Dyck PJ B Parisi JE Mer G Smith DI Dyck PJ Mutations in DNMT1 cause hereditary sensory neuropathy with dementia and hearing loss Nat Genet 2011 14 595 600 10.1038/ng.830 21532572 PMC3102765

[B16] Winkelmann J Lin L Schormair B Kornum BR Faraco J Plazzi G Melberg A Cornelio F Urban AE Pizza F Poli F Grubert F Wieland T Graf E Hallmayer J Strom TM Mignot E Mutations in DNMT1 cause autosomal dominant cerebellar ataxia, deafness and narcolepsy Hum Mol Genet 2012 14 2205 2210 10.1093/hmg/dds035 22328086 PMC3465691

[B17] Kullmann K Deryal M Ong MF Schmidt W Mahlknecht U DNMT1 genetic polymorphisms affect breast cancer risk in the central European Caucasian population Clin Epigenetics 2013 14 7 10.1186/1868-7083-5-7 23638630 PMC3646668

[B18] Veldic M Guidotti A Maloku E Davis JM Costa E In psychosis, cortical interneurons overexpress DNA-methyltransferase 1 Proc Natl Acad Sci U S A 2005 14 2152 2157 10.1073/pnas.0409665102 15684088 PMC548582

[B19] Patra SK Patra A Zhao H Dahiya R DNA methyltransferase and demethylase in human prostate cancer Mol Carcinog 2002 14 163 171 10.1002/mc.10033 11870882

[B20] Stopka T Zakova D Fuchs O Kubrova O Blafkova J Jelinek J Necas E Zivny J Chromatin remodeling gene SMARCA5 is dysregulated in primitive hematopoietic cells of acute leukemia Leukemia 2000 14 1247 1252 10.1038/sj.leu.2401807 10914549

[B21] Lu X Meng X Morris CA Keating MT A novel human gene, WSTF, is deleted in Williams syndrome Genomics 1998 14 241 249 10.1006/geno.1998.5578 9828126

[B22] Ceol CJ Houvras Y Jane-Valbuena J Bilodeau S Orlando DA Battisti V Fritsch L Lin WM Hollmann TJ Ferré F Bourque C Burke CJ Turner L Uong A Johnson LA Beroukhim R Mermel CH Loda M Ait-Si-Ali S Garraway LA Young RA Zon LI The histone methyltransferase SETDB1 is recurrently amplified in melanoma and accelerates its onset Nature 2011 14 513 517 10.1038/nature09806 21430779 PMC3348545

[B23] Xu GL Bestor TH Bourc'his D Hsieh CL Tommerup N Bugge M Hulten M Qu X Russo JJ Viegas-Pequignot E Chromosome instability and immunodeficiency syndrome caused by mutations in a DNA methyltransferase gene Nature 1999 14 187 191 10.1038/46052 10647011

[B24] Wang H Zhao A Chen L Zhong X Liao J Gao M Cai M Lee DH Li J Chowdhury D Yang YG Pfeifer GP Yen Y Xu X Human RIF1 encodes an anti-apoptotic factor required for DNA repair Carcinogenesis 2009 14 1314 1319 10.1093/carcin/bgp136 19483192 PMC2718077

[B25] Andersen CL Christensen LL Thorsen K Schepeler T Sorensen FB Verspaget HW Simon R Kruhoffer M Aaltonen LA Laurberg S Orntoft TF Dysregulation of the transcription factors SOX4, CBFB and SMARCC1 correlates with outcome of colorectal cancer Br J Cancer 2009 14 511 523 10.1038/sj.bjc.6604884 19156145 PMC2658541

[B26] DelBove J Rosson G Strobeck M Chen J Archer TK Wang W Knudsen ES Weissman BE Identification of a core member of the SWI/SNF complex, BAF155/SMARCC1, as a human tumor suppressor gene Epigenetics 2011 14 1444 1453 10.4161/epi.6.12.18492 22139574 PMC3256333

[B27] Varela I Tarpey P Raine K Huang D Ong CK Stephens P Davies H Jones D Lin ML Teague J Bignell G Butler A Cho J Dalgliesh GL Galappaththige D Greenman C Hardy C Jia M Latimer C Lau KW Marshall J McLaren S Menzies A Mudie L Stebbings L Largaespada DA Wessels LF Richard S Kahnoski RJ Anema J Tuveson DA Exome sequencing identifies frequent mutation of the SWI/SNF complex gene PBRM1 in renal carcinoma Nature 2011 14 539 542 10.1038/nature09639 21248752 PMC3030920

[B28] Tsurusaki Y Okamoto N Ohashi H Kosho T Imai Y Hibi-Ko Y Kaname T Naritomi K Kawame H Wakui K Fukushima Y Homma T Kato M Hiraki Y Yamagata T Yano S Mizuno S Sakazume S Ishii T Nagai T Shiina M Ogata K Ohta T Niikawa N Miyatake S Okada I Mizuguchi T Doi H Saitsu H Miyake N Matsumoto N Mutations affecting components of the SWI/SNF complex cause Coffin-Siris syndrome Nat Genet 2012 14 376 378 10.1038/ng.2219 22426308

[B29] Severinsen JE Bjarkam CR Kiaer-Larsen S Olsen IM Nielsen MM Blechingberg J Nielsen AL Holm IE Foldager L Young BD Muir WJ Blackwood DH Corydon TJ Mors O Børglum AD Evidence implicating BRD1 with brain development and susceptibility to both schizophrenia and bipolar affective disorder Mol Psychiatry 2006 14 1126 1138 10.1038/sj.mp.4001885 16924267

[B30] Arnold CN Barnes MJ Berger M Blasius AL Brandl K Croker B Crozat K Du X Eidenschenk C Georgel P Hoebe K Huang H Jiang Z Krebs P La Vine D Li X Lyon S Moresco EM Murray AR Popkin DL Rutschmann S Siggs OM Smart NG Sun L Tabeta K Webster V Tomisato W Won S Xia Y Xiao N Beutler B ENU-induced phenovariance in mice: inferences from 587 mutations BMC Res Notes 2012 14 577 10.1186/1756-0500-5-577 23095377 PMC3532239

[B31] Keays DA Clark TG Campbell TG Broxholme J Valdar W Estimating the number of coding mutations in genotypic and phenotypic driven N-ethyl-N-nitrosourea (ENU) screens: revisited Mamm Genome 2007 14 123 124 10.1007/s00335-006-0065-z 17347895

[B32] Tachibana M Ueda J Fukuda M Takeda N Ohta T Iwanari H Sakihama T Kodama T Hamakubo T Shinkai Y Histone methyltransferases G9a and GLP form heteromeric complexes and are both crucial for methylation of euchromatin at H3-K9 Genes Dev 2005 14 815 826 10.1101/gad.1284005 15774718 PMC1074319

[B33] Ueda J Tachibana M Ikura T Shinkai Y Zinc finger protein Wiz links G9a/GLP histone methyltransferases to the co-repressor molecule CtBP J Biol Chem 2006 14 20120 20128 10.1074/jbc.M603087200 16702210

[B34] Chapman JR Barral P Vannier JB Borel V Steger M Tomas-Loba A Sartori AA Adams IR Batista FD Boulton SJ RIF1 is essential for 53BP1-dependent nonhomologous end joining and suppression of DNA double-strand break resection Mol Cell 2013 14 858 871 10.1016/j.molcel.2013.01.002 23333305 PMC3594748

[B35] Di Virgilio M Callen E Yamane A Zhang W Jankovic M Gitlin AD Feldhahn N Resch W Oliveira TY Chait BT Nussenzweig A Casellas R Robbiani DF Nussenzweig MC Rif1 prevents resection of DNA breaks and promotes immunoglobulin class switching Science 2013 14 711 715 10.1126/science.1230624 23306439 PMC3815530

[B36] Escribano-Díaz C Orthwein A Fradet-Turcotte A Xing M Young JT Tkáč J Cook MA Rosebrock AP Munro M Canny MD Xu D Durocher D A cell cycle-dependent regulatory circuit composed of 53BP1-RIF1 and BRCA1-CtIP controls DNA repair pathway choice Mol Cell 2013 14 872 883 10.1016/j.molcel.2013.01.001 23333306

[B37] Zimmermann M Lottersberger F Buonomo SB Sfeir A de Lange T 53BP1 regulates DSB repair using Rif1 to control 5’ end resection Science 2013 14 700 704 10.1126/science.1231573 23306437 PMC3664841

[B38] Cornacchia D Dileep V Quivy JP Foti R Tili F Santarella-Mellwig R Antony C Almouzni G Gilbert DM Buonomo SB Mouse Rif1 is a key regulator of the replication-timing programme in mammalian cells EMBO J 2012 14 3678 3690 10.1038/emboj.2012.214 22850673 PMC3442270

[B39] Yamazaki S Ishii A Kanoh Y Oda M Nishito Y Masai H Rif1 regulates the replication timing domains on the human genome EMBO J 2012 14 3667 3677 10.1038/emboj.2012.180 22850674 PMC3442267

[B40] Makela TP Hellsten E Vesa J Hirvonen H Palotie A Peltonen L Alitalo K The rearranged L-myc fusion gene (RLF) encodes a Zn-15 related zinc finger protein Oncogene 1995 14 2699 2704 8545128

[B41] Waterland RA Assessing the effects of high methionine intake on DNA methylation J Nutr 2006 14 1706S 1710S 16702343 10.1093/jn/136.6.1706S

[B42] Morgan HD Sutherland HG Martin DI Whitelaw E Epigenetic inheritance at the agouti locus in the mouse Nat Genet 1999 14 314 318 10.1038/15490 10545949

[B43] Blewitt ME Vickaryous NK Paldi A Koseki H Whitelaw E Dynamic reprogramming of DNA methylation at an epigenetically sensitive allele in mice PLoS Genet 2006 14 e49 10.1371/journal.pgen.0020049 16604157 PMC1428789

[B44] Gaudet F Rideout WM 3rd Meissner A Dausman J Leonhardt H Jaenisch R Dnmt1 expression in pre- and postimplantation embryogenesis and the maintenance of IAP silencing Mol Cell Biol 2004 14 1640 1648 10.1128/MCB.24.4.1640-1648.2004 14749379 PMC344181

[B45] Mishima Y Miyagi S Saraya A Negishi M Endoh M Endo TA Toyoda T Shinga J Katsumoto T Chiba T Yamaguchi N Kitabayashi I Koseki H Iwama A The Hbo1-Brd1/Brpf2 complex is responsible for global acetylation of H3K14 and required for fetal liver erythropoiesis Blood 2011 14 2443 2453 10.1182/blood-2011-01-331892 21753189

[B46] Sharif J Muto M Takebayashi S Suetake I Iwamatsu A Endo TA Shinga J Mizutani-Koseki Y Toyoda T Okamura K Tajima S Mitsuya K Okano M Koseki H The SRA protein Np95 mediates epigenetic inheritance by recruiting Dnmt1 to methylated DNA Nature 2007 14 908 912 10.1038/nature06397 17994007

[B47] Rothbart SB Dickson BM Ong MS Krajewski K Houliston S Kireev DB Arrowsmith CH Strahl BD Multivalent histone engagement by the linked tandem Tudor and PHD domains of UHRF1 is required for the epigenetic inheritance of DNA methylation Genes Dev 2013 14 1288 1298 10.1101/gad.220467.113 23752590 PMC3690401

[B48] Buonomo SB Wu Y Ferguson D de Lange T Mammalian Rif1 contributes to replication stress survival and homology-directed repair J Cell Biol 2009 14 385 398 10.1083/jcb.200902039 19948482 PMC2779251

[B49] Dodge JE Kang YK Beppu H Lei H Li E Histone H3-K9 methyltransferase ESET is essential for early development Mol Cell Biol 2004 14 2478 2486 10.1128/MCB.24.6.2478-2486.2004 14993285 PMC355869

[B50] Kim JK Huh SO Choi H Lee KS Shin D Lee C Nam JS Kim H Chung H Lee HW Park SD Seong RH Srg3, a mouse homolog of yeast SWI3, is essential for early embryogenesis and involved in brain development Mol Cell Biol 2001 14 7787 7795 10.1128/MCB.21.22.7787-7795.2001 11604513 PMC99948

[B51] Bultman S Gebuhr T Yee D La Mantia C Nicholson J Gilliam A Randazzo F Metzger D Chambon P Crabtree G Magnuson T A Brg1 null mutation in the mouse reveals functional differences among mammalian SWI/SNF complexes Mol Cell 2000 14 1287 1295 10.1016/S1097-2765(00)00127-1 11163203

[B52] Messerschmidt DM de Vries W Ito M Solter D Ferguson-Smith A Knowles BB Trim28 is required for epigenetic stability during mouse oocyte to embryo transition Science 2012 14 1499 1502 10.1126/science.1216154 22442485

[B53] Pujadas E Feinberg AP Regulated noise in the epigenetic landscape of development and disease Cell 2012 14 1123 1131 10.1016/j.cell.2012.02.045 22424224 PMC3488344

[B54] Allen ND Norris ML Surani MA Epigenetic control of transgene expression and imprinting by genotype-specific modifiers Cell 1990 14 853 861 10.1016/0092-8674(90)90195-K 2111735

[B55] Sutherland HG Kearns M Morgan HD Headley AP Morris C Martin DI Whitelaw E Reactivation of heritably silenced gene expression in mice Mamm Genome 2000 14 347 355 10.1007/s003350010066 10790532

[B56] Rakyan VK Chong S Champ ME Cuthbert PC Morgan HD Luu KV Whitelaw E Transgenerational inheritance of epigenetic states at the murine Axin(Fu) allele occurs after maternal and paternal transmission Proc Natl Acad Sci U S A 2003 14 2538 2543 10.1073/pnas.0436776100 12601169 PMC151376

[B57] Li H Durbin R Fast and accurate short read alignment with Burrows-Wheeler transform Bioinformatics 2009 14 1754 1760 10.1093/bioinformatics/btp324 19451168 PMC2705234

[B58] Li H Durbin R Fast and accurate long-read alignment with Burrows-Wheeler transform Bioinformatics 2010 14 589 595 10.1093/bioinformatics/btp698 20080505 PMC2828108

[B59] Li H Handsaker B Wysoker A Fennell T Ruan J Homer N Marth G Abecasis G Durbin R Genome Project Data Processing S: The Sequence Alignment/Map format and SAMtools Bioinformatics 2009 14 2078 2079 10.1093/bioinformatics/btp352 19505943 PMC2723002

[B60] Picardhttp://picard.sourceforge.net

[B61] Koboldt DC Zhang Q Larson DE Shen D McLellan MD Lin L Miller CA Mardis ER Ding L Wilson RK VarScan 2: somatic mutation and copy number alteration discovery in cancer by exome sequencing Genome Res 2012 14 568 576 10.1101/gr.129684.111 22300766 PMC3290792

[B62] Bock C Reither S Mikeska T Paulsen M Walter J Lengauer T BiQ Analyzer: visualization and quality control for DNA methylation data from bisulfite sequencing Bioinformatics 2005 14 4067 4068 10.1093/bioinformatics/bti652 16141249

[B63] Divina P Kvitkovicova A Buratti E Vorechovsky I Ab initio prediction of mutation-induced cryptic splice-site activation and exon skipping Eur J Hum Genet 2009 14 759 765 10.1038/ejhg.2008.257 19142208 PMC2947103

