# Supplementary figures and images for: An ENU mutagenesis screen identifies novel and known genes involved in epigenetic processes in the mouse
Source: Genome Biol. 2013 Sep 11;14(9):R96. doi: 10.1186/gb-2013-14-9-r96 (PMC4053835; doi:10.1186/gb-2013-14-9-r96)

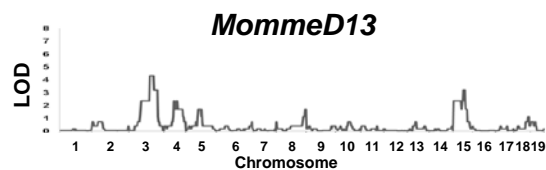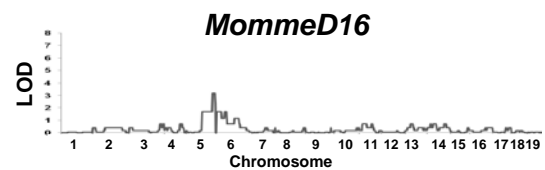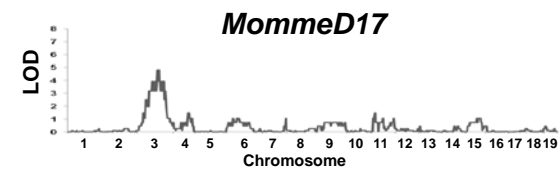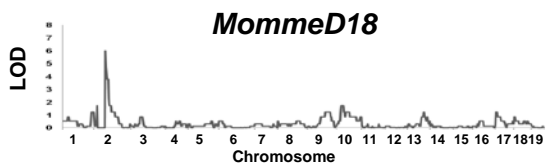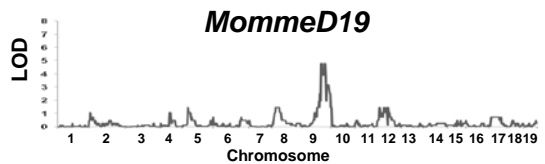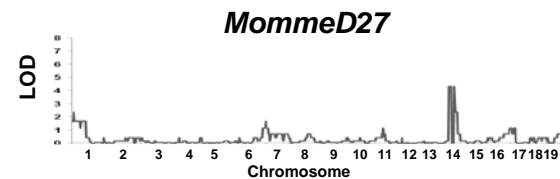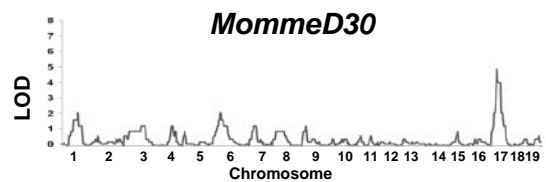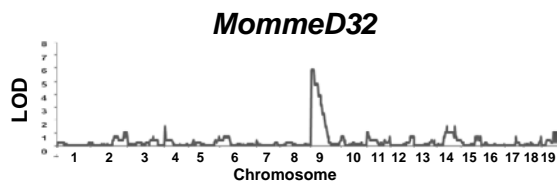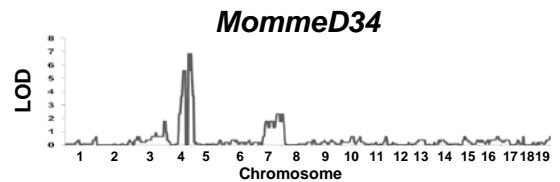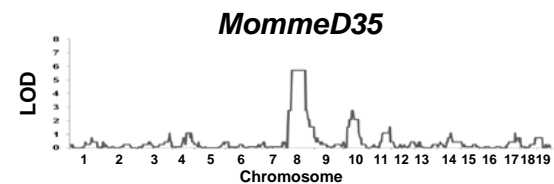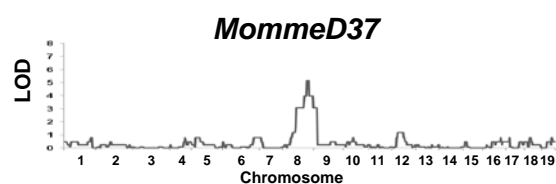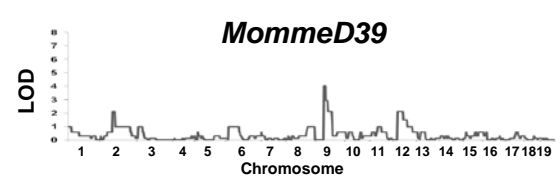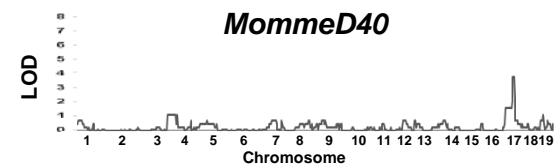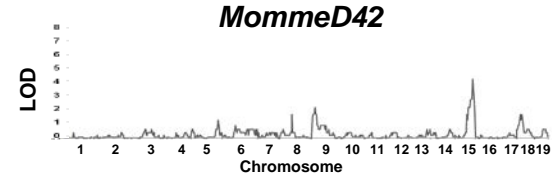

**Additional File 1**

Supplement: Additional file 1 — Linked intervals. Manhattan plots showing linked intervals identified by Illumina GoldenGate SNP genotyping analysis. The x-axis represents the chromosomes and the y-axis is the LOD score. Peaks with a LOD score of 3 or higher are considered significant. [file gb-2013-14-9-r96-S1.pdf]

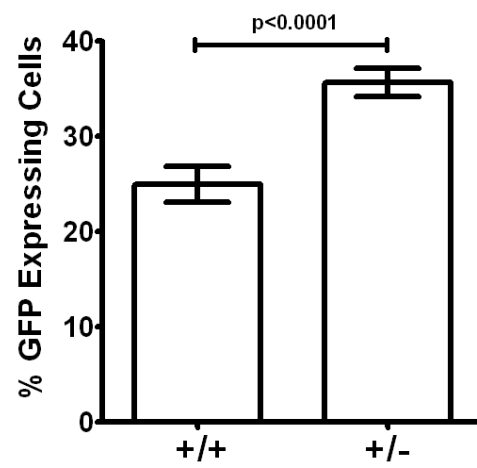

Supplement: Additional file 4 — GFP expression in offspring of a Rif1GTheterozygote crossed to Line3C. A Rif1 gene trap allele (Rif1GT) had a similar effect on transgene expression as that observed with the MommeD18 mutation, increasing the percentage of expressing cells in mice heterozygous for the gene-trap allele. [file gb-2013-14-9-r96-S4.pdf]

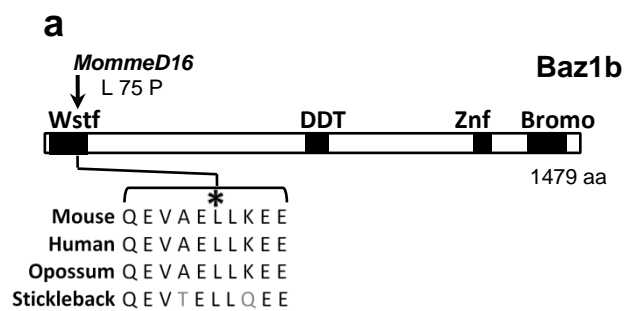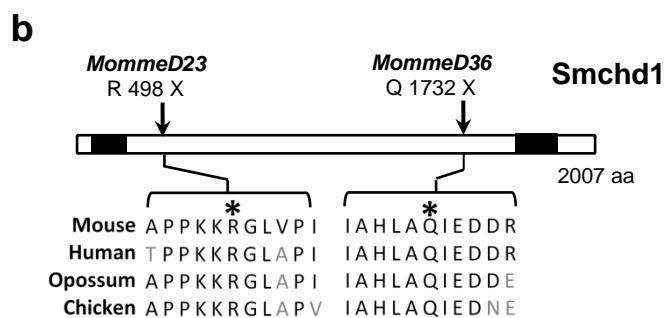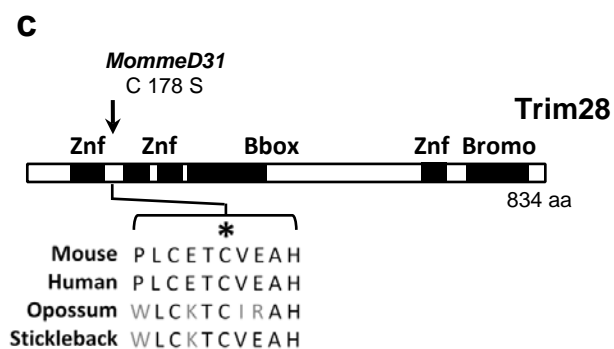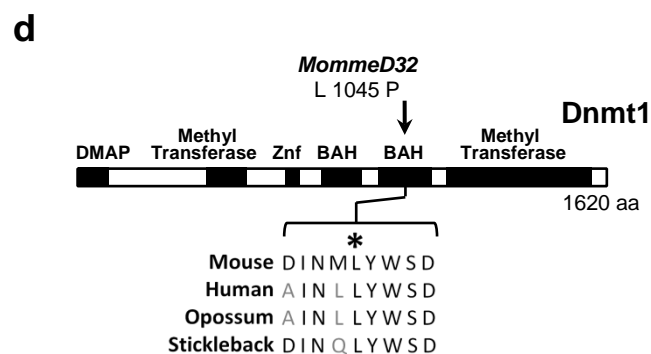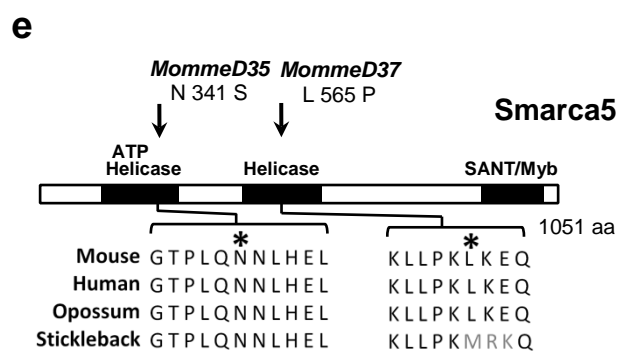

Supplement: Additional file 5 — Novel mutant alleles of Baz1b, Smchd1, Trim28, Dnmt1 and Smarca5. (a)MommeD16 carries a point mutation resulting in a non-conservative amino acid change in the Wstf domain of Baz1b. (b)MommeD23 and MommeD36 carry point mutations in Smchd1. Both mutations introduce premature stop codons in the Smchd1 protein. (c)MommeD31 carries a point mutation in Trim28 that results in an amino acid change in a highly conserved zinc finger domain. (d)MommeD32 carries a point mutation that results in an amino acid change in the BAH domain of Dnmt1. (e)MommeD35 and MommeD37 carry mutations that result in amino acid changes in highly conserved domains of the Smarca5 protein. [file gb-2013-14-9-r96-S5.pdf]

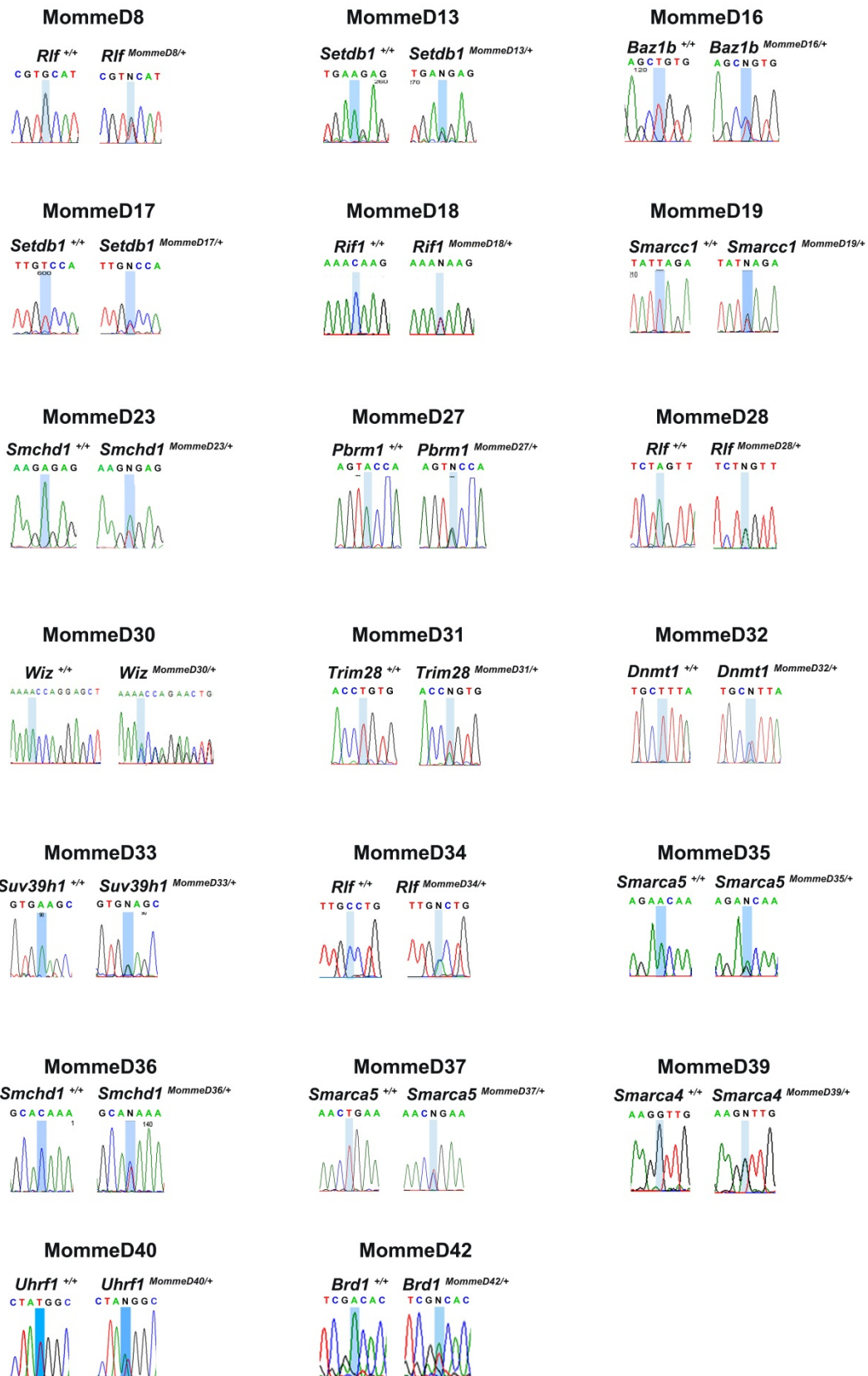

Supplement: Additional file 9 — Sanger traces. [file gb-2013-14-9-r96-S9.pdf]
