# Supplementary material for: An ENU mutagenesis screen identifies novel and known genes involved in epigenetic processes in the mouse
Source: Genome Biol. 2013 Sep 11;14(9):R96. doi: 10.1186/gb-2013-14-9-r96 (PMC4053835; doi:10.1186/gb-2013-14-9-r96)
Supplement: Additional file 2 — Flow cytometric profiles of the MommeDs. (a) Representative flow cytometry profiles show the percentage of GFP-expressing erythrocytes from wild-type and heterozygous mutant littermates (n = 3). The x-axis represents the erythrocyte fluorescence on a logarithmic scale and the y-axis is the number of cells detected at each fluorescence level. (b) Percentage of GFP-expressing cells in wild-type, heterozygous mutant and homozygous mutant (where viable) mice at three weeks of age (mean ± standard error of the mean). [file gb-2013-14-9-r96-S2.pdf]

a

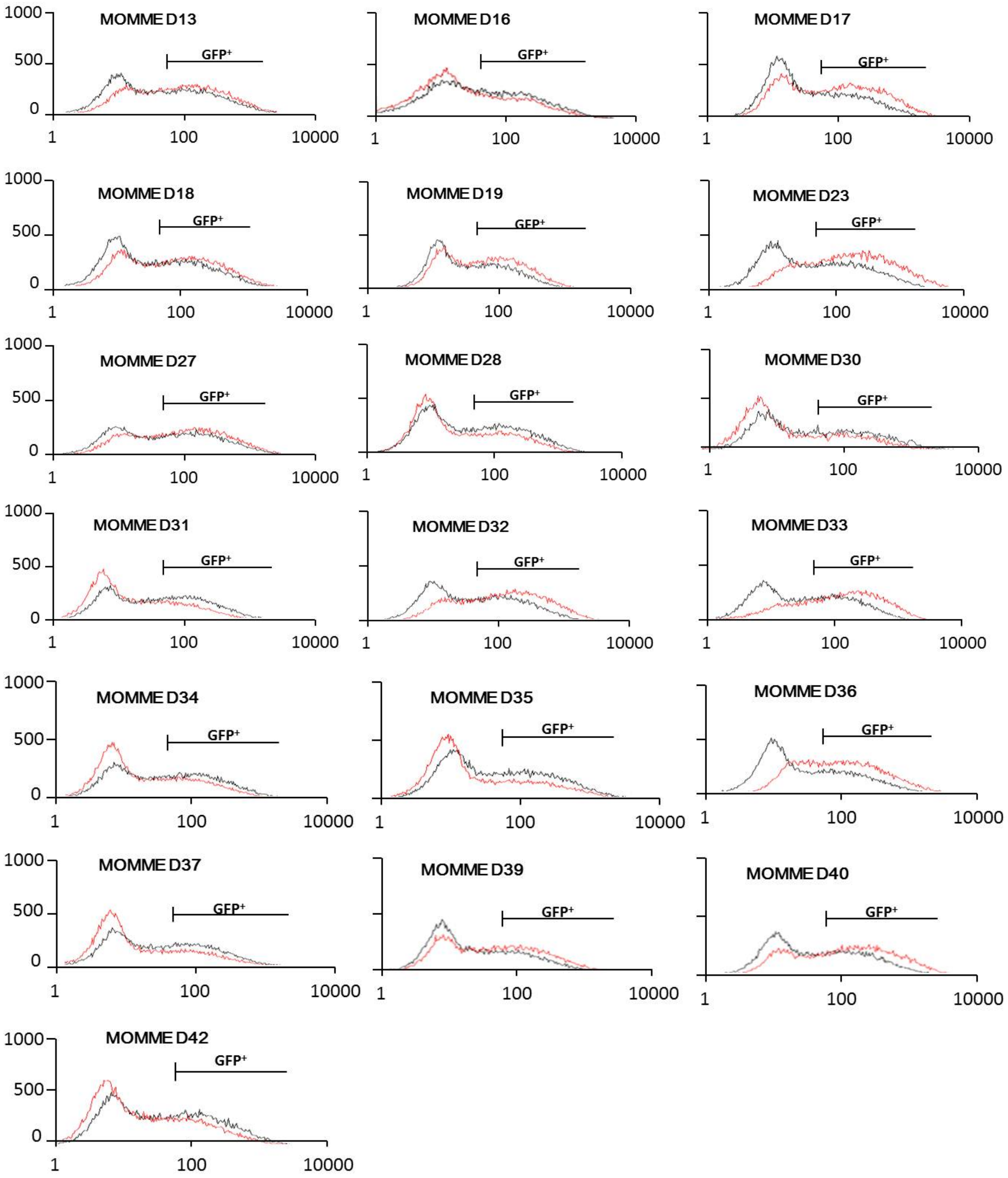

b

Suppressors of Variegation - Percentage GFP Expressing Cells				
**Name**	**Gene**	**+/+**	**-/+**	**-/-**
MommeD13	*Setdb1*	53 ± 4	67 ± 3	
MommeD17	*Setdb1*	54 ± 4	65 ± 4	80 ± 4
MommeD18	*Rif1*	54 ± 2	65 ± 2	83 ± 2
MommeD19	*Smarcc1*	54 ± 2	65 ± 5	
MommeD23	*Smchd1*	59 ± 2	88 ± 2	98 ± 2
MommeD27	*Pbrm1*	60 ± 4	71 ± 3	
MommeD32	*Dnmt1*	59 ± 4	80 ± 3	
MommeD33	*Suv39h1*	60 ± 2	71 ± 4#	
MommeD36	*Smchd1*	57 ± 2	84 ± 2	
MommeD39	*Smarca4*	59 ± 2	71 ± 2	
MommeD40	*Uhrf1*	58 ± 3	75 ± 1	

Enhancers of Variegation - Percentage GFP Expressing Cells				
**Name**	**Gene**	**+/+**	**-/+**	**-/-**
MommeD8	*Rlf*	54 ± 4	41 ± 4	15 ± 4
MommeD16	*Baz1b*	57 ± 2	42 ± 2	20 ± 4
MommeD28	*Rlf*	59 ± 3	44 ± 3	7*
MommeD30	*Wiz*	59 ± 3	46 ± 3	
MommeD31	*Trim 28*	58 ± 3	46 ± 2	
MommeD34	*Rlf*	52 ± 6	39 ± 5	11 ± 2
MommeD35	*Smarca5*	58 ± 1	41 ± 3	
MommeD37	*Smarca5*	54 ± 3	32 ± 2	
MommeD42	*Brd1*	56 ± 2	44 ± 3	

* Data from the single viable *D28/D28* mutant
# % GFP expressing cells in hemizygous male *D33/0* mice

Additional File 2
