# Supplementary material for: An ENU mutagenesis screen identifies novel and known genes involved in epigenetic processes in the mouse
Source: Genome Biol. 2013 Sep 11;14(9):R96. doi: 10.1186/gb-2013-14-9-r96 (PMC4053835; doi:10.1186/gb-2013-14-9-r96)
Supplement: Additional file 3 — Mapping interval for MommeD30. (a)MommeD30 was produced in the FVB/NJ strain of mice and mapped by crossing twice onto Line3C in a C57BL/6J background. The results of the genotype for seven SNP markers and one microsatellite marker surrounding the linked interval are shown. The number of mice classified into each haplotype is shown on top. Our estimate of the linked interval is between rs29539305 and rs33446195 on chromosome 17 (highlighted). (b) List of genes in the MommeD30 linked interval on chromosome 17. The Mus musculus Ensembl database (release 37) was used to export a list of transcripts (protein coding and non-coding RNAs) within the 1.9 Mbp MommeD30 interval. [file gb-2013-14-9-r96-S3.pdf]

(a)

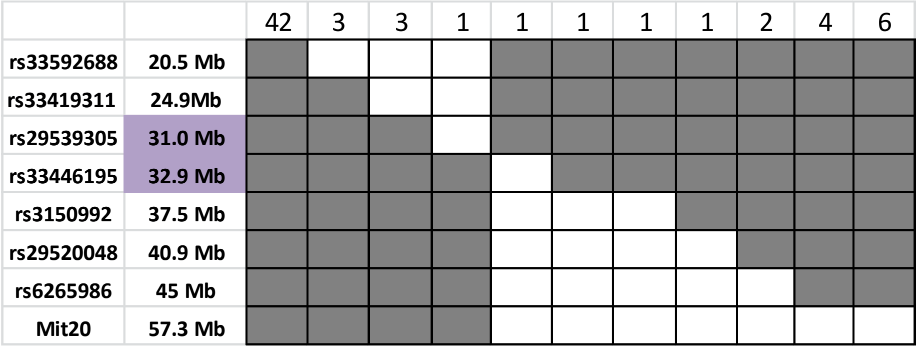

(b)

Gene Start (bp)	Associated Gene Name	Description
30761354	Dnahc8	dynein, axonemal, heavy chain 8 Gene [Source:MGI Symbol;Acc:MGI:107714]
31011751	Gm9937	predicted gene 9937 Gene [Source:MGI Symbol;Acc:MGI:3642051]
31038812	Glp1r	glucagon-like peptide 1 receptor Gene [Source:MGI Symbol;Acc:MGI:99571]
31060691	Gm10503	predicted gene 10503 Gene [Source:MGI Symbol;Acc:MGI:3642784]
31091624	Umodl1	uromodulin-like 1 Gene [Source:MGI Symbol;Acc:MGI:1929785]
31194643	Abcg1	ATP-binding cassette, sub-family G (WHITE), member 1 Gene [Source:MGI (curated)
31196549	5S_rRNA	5S ribosomal RNA [Source:RFAM;Acc:RF00001]
31262251	Tff3	trefoil factor 3, intestinal Gene [Source:MGI (curated);Acc:MGI:104638]
31277994	Tff2	trefoil factor 2 (spasmolytic protein 1) Gene [Source:MGI (curated);Acc:MGI:1306805
31289697	RP23-418C19.4	
31298341	Tff1	trefoil factor 1 Gene [Source:MGI (curated);Acc:MGI:88135]
31316217	Tmprss3	transmembrane protease, serine 3 Gene [Source:MGI (curated);Acc:MGI:2155445]
31344818	Ubash3a	ubiquitin associated and SH3 domain containing, A Gene [Source:MGI (curated);Acc
31391969	Rsph1	radial spoke head 1 homolog (Chlamydomonas) Gene [Source:MGI Symbol;Acc:MGI
31433702	Slc37a1	solute carrier family 37 (glycerol-3-phosphate transporter), member 1 Gene [Source:M
31523179	Pde9a	phosphodiesterase 9A Gene [Source:MGI (curated);Acc:MGI:1277179]
31570894	Gm9902	predicted gene 9902 Gene [Source:MGI Symbol;Acc:MGI:3642122]
31632569	Wdr4	WD repeat domain 4 Gene [Source:MGI Symbol;Acc:MGI:1889002]
31657120	Ndufv3	NADH dehydrogenase (ubiquinone) flavoprotein 3 Gene [Source:MGI Symbol;Acc:MG
31694071	RP23-106N22.1	
31701746	Pknox1	Pbx/knotted 1 homeobox Gene [Source:MGI Symbol;Acc:MGI:1201409]
31749568	Cbs	cystathionine beta-synthase Gene [Source:MGI (curated);Acc:MGI:88285]
31784027	U2af1	U2 small nuclear ribonucleoprotein auxiliary factor (U2AF) 1 Gene [Source:MGI Symb
31814878	Cryaa	crystallin, alpha A Gene [Source:MGI Symbol;Acc:MGI:88515]
31981193	Sik1	salt inducible kinase 1 Gene [Source:MGI Symbol;Acc:MGI:104754]
32081714	Hsf2bp	heat shock transcription factor 2 binding protein Gene [Source:MGI (curated);Acc:MG
32164883	7SK	7SK RNA [Source:RFAM;Acc:RF00100]
32173045	Rrp1b	ribosomal RNA processing 1 homolog B (S. cerevisiae) Gene [Source:MGI (curated);
32213028	RP23-451J17.2	pyridoxal (pyridoxine, vitamin B6) kinase, pseudogene (Pdxk-ps), non-coding RNA [S
32257765	Notch3	Notch gene homolog 3 (Drosophila) Gene [Source:MGI (curated);Acc:MGI:99460]
32320715	Ephx3	epoxide hydrolase 3 Gene [Source:MGI (curated);Acc:MGI:1919182]
32333219	Brd4	bromodomain containing 4 Gene [Source:MGI (curated);Acc:MGI:1888520]
32440621	Akap8	A kinase (PRKA) anchor protein 8 Gene [Source:MGI Symbol;Acc:MGI:1928488]
32458370	Akap8l	A kinase (PRKA) anchor protein 8-like Gene [Source:MGI Symbol;Acc:MGI:1860606
32491011	Wiz	widely-interspaced zinc finger motifs Gene [Source:MGI Symbol;Acc:MGI:1332638]
32525956	CT485616.1	
32527604	Rasal3	RAS protein activator like 3 Gene [Source:MGI (curated);Acc:MGI:2444128]
32539978	A530088E08Rik	RIKEN cDNA A530088E08 gene Gene [Source:MGI Symbol;Acc:MGI:3603459]
32550306	Pglyrp2	peptidoglycan recognition protein 2 Gene [Source:MGI Symbol;Acc:MGI:1928099]
32589668	Cyp4f39	cytochrome P450, family 4, subfamily f, polypeptide 39 Gene [Source:MGI Symbol;A
32673545	Cyp4f16	cytochrome P450, family 4, subfamily f, polypeptide 16 Gene [Source:MGI Symbol;A
32758264	Gm9705	predicted gene 9705 Gene [Source:MGI Symbol;Acc:MGI:3780112]
32822624	Cyp4f15	cytochrome P450, family 4, subfamily f, polypeptide 15 Gene [Source:MGI Symbol;A
32910210	Zfp871	zinc finger protein 871 Gene [Source:MGI Symbol;Acc:MGI:1921793]
32933958	Zfp811	zinc finger protein 811 Gene [Source:MGI Symbol;Acc:MGI:2682944]
32937512	U1	U1 spliceosomal RNA [Source:RFAM;Acc:RF00003]
32941812	5S_rRNA	5S ribosomal RNA [Source:RFAM;Acc:RF00001]
32947089	CT485613.2	
32952400	Zfp799	zinc finger protein 799 Gene [Source:MGI Symbol;Acc:MGI:2443934]
33016173	Zfp870	zinc finger protein 870 Gene [Source:MGI Symbol;Acc:MGI:3029586]

Additional File 6
