# Supplementary material for: An ENU mutagenesis screen identifies novel and known genes involved in epigenetic processes in the mouse
Source: Genome Biol. 2013 Sep 11;14(9):R96. doi: 10.1186/gb-2013-14-9-r96 (PMC4053835; doi:10.1186/gb-2013-14-9-r96)
Supplement: Additional file 6 — Embryonic development in MommeD mutants. Tabulated data shows the number of observed mice and in brackets the percentage of total. (a) Intercrosses of Pbrm1MommeD27 mice produce no homozygous offspring at three weeks. (b)Brd1MommeD42 homozygous mice are embryonic lethal around 10.5 dpc. (c) Mice carrying mutations in Suv39h1MommeD33 are viable at three weeks. (d)Uhrf1MommeD40 homozygotes are embryonic lethal around 10.5 dpc. (e) Some homozygous Baz1bMommeD16 mice were obtained at three weeks but less than expected. (f) No (Smchd1MommeD36) or few (Smchd1MommeD23) homozygous individuals were recovered from intercrosses. The Smchd1MommeD23 homozygotes that survived were males. (g)Trim28MommeD31 homozygous mice are embryonic lethal prior to 10.5 dpc. (h) Mice homozygous for the Dnmt1MommeD32 mutation die around 10.5 dpc. (i) Intercrosses of Smarca5MommeD35 and Smarca5MommeD37 produced no homozygous offspring at three weeks. Timed matings show that survival of homozygous mutants at 12.5 to 14.5 dpc was rare in Smarca5MommeD35 mutants. In Smarca5MommeD37 mutants homozygous embryonic death occurred prior to 14.5 dpc. [file gb-2013-14-9-r96-S6.pdf]

a

Pbrm1^{MommeD27} Intercross

Age	+/+	D27/+	D27/D27	Empty Decidua
**14.5 dpc**	11 (18)	27 (45)	15 (25)*	7 (12)
**3 weeks**	19 (35)	35 (65)	0 (0)	na

*4 embryos were smaller than littermates, 3 of these were pale.

b

Brd1^{MommeD42} Intercross

Age	+/+	D42/+	D42/D42
**10.5 dpc**	4 (14)	16 (55)	9 (31)
**14.5 dpc**	9 (50)	9 (50)	0 (0)
**3 weeks**	14 (41)	20 (59)	0 (0)

c

Suv39h1^{MommeD33} Intercross

	♂		♀	
**Age**	**+/0**	**D33/0**	**D33/+**	**D33/D33**
**3 weeks**	34 (25)	34 (25)	33 (24)	37 (26)

d

Uhrf1^{MommeD40} Intercross

Age	+/+	D40/+	D40/D40	Empty Decidua
**10.5 – 11.5 dpc**	6 (26)	11 (48)	3 (13)	3 (13)
**3 weeks**	18 (33)	37 (67)	0 (0)	na

e

Baz1b^{MommeD16} Intercross

Age	+/+	D16/+	D16/D16
**3 weeks**	17 (25)	47 (70)	3 (4)

f

Smchd1 Intercross

3 weeks	+/+	+/-	-/-
MD23$^{-/+}$ ***x MD23***$^{-/+}$	54 (45)	62 (52)	4 (3)
MD36$^{-/+}$ ***x MD36***$^{-/+}$	17 (35)	32 (65)	0 (0)
MD23$^{-/+}$***x MD36***$^{-/+}$	13 (36)	23 (64)	0 (0)

g

Trim28^{MommeD31} Intercross

Age	+/+	D31/+	D31/D31	Empty Decidua
**10.5 dpc**	7 (29)	12 (50)	0 (0)	5 (21)

h

Dnmt1^{MommeD32} Intercross

Age	+/+	D32/+	D32/D32	Empty Decidua
**10.5 dpc**	8 (22)	13 (36)	4 (11)*	11 (30)
**3 weeks**	25 (31)	55 (69)	0 (0)	na

*4/4 homozygous mutants were developmentally abnormal

i

Smarca5 Intercross

Cross	Age	+/+	+/-	-/-	Empty Decidua
D35$^{-/+}$ ***x D35***$^{-/+}$	12.5-14.5 dpc	11 (34)	15 (47)	6 (19)	0
D35$^{-/+}$ ***x D35***$^{-/+}$	3 weeks	16 (32)	34 (68)	0 (0)	na
D37$^{-/+}$ ***x D37***$^{-/+}$	14.5 dpc	14 (24)	22 (37)	0 (0)	23 (39)
D37$^{-/+}$ ***xD37***$^{-/+}$	3 weeks	20 (36)	36 (64)	0 (0)	na

Additional File 4
